# Cell Surface Modification-Mediated Primary Intestinal Epithelial Cell Culture Platforms for Assessing Host–Microbiota Interactions

**DOI:** 10.34133/bmr.0004

**Published:** 2024-01-25

**Authors:** Panida Sittipo, Laurensia Danis Anggradita, Hyunbum Kim, Chanyoung Lee, Nathaniel S. Hwang, Yun Kyung Lee, Yongsung Hwang

**Affiliations:** ^1^ Soonchunhyang Institute of Medi-bio Science (SIMS), Soonchunhyang University, Cheonan-si, Chungnam-do 31151, Republic of Korea.; ^2^ Department of Integrated Biomedical Science, Soonchunhyang University, Asan-si, Chungnam-do 31538, Republic of Korea.; ^3^ School of Chemical and Biological Engineering, Institute of Chemical Processes, Seoul National University, Seoul 08826, Republic of Korea.; ^4^ Bio-MAX/N-Bio Institute, Institute of Bio-Engineering, Seoul National University, Seoul 08826, Republic of Korea.; ^5^ Institute of Engineering Research, Seoul National University, Seoul 08826, Republic of Korea.

## Abstract

**Background:** Intestinal epithelial cells (IECs) play a crucial role in regulating the symbiotic relationship between the host and the gut microbiota, thereby allowing them to modulate barrier function, mucus production, and aberrant inflammation. Despite their importance, establishing an effective ex vivo culture method for supporting the prolonged survival and function of primary IECs remains challenging. Here, we aim to develop a novel strategy to support the long-term survival and function of primary IECs in response to gut microbiota by employing mild reduction of disulfides on the IEC surface proteins with tris(2-carboxyethyl)phosphine. **Methods:** Recognizing the crucial role of fibroblast-IEC crosstalk, we employed a cell surface modification strategy, establishing layer-to-layer contacts between fibroblasts and IECs. This involved combining negatively charged chondroitin sulfate on cell surfaces with a positively charged chitosan thin film between cells, enabling direct intercellular transfer. Validation included assessments of cell viability, efficiency of dye transfer, and IEC function upon lipopolysaccharide (LPS) treatment. **Results:** Our findings revealed that the layer-by-layer co-culture platform effectively facilitates the transfer of small molecules through gap junctions, providing vital support for the viability and function of primary IECs from both the small intestine and colon for up to 5 days, as evident by the expression of E-cadherin and Villin. Upon LPS treatment, these IECs exhibited a down-regulation of *Villin* and tight junction genes, such as *E-cadherin* and *Zonula Occludens-1*, when compared to their nontreated counterparts. Furthermore, the transcription level of *Lysozyme* exhibited an increase, while *Mucin 2* showed a decrease in response to LPS, indicating responsiveness to bacterial molecules. **Conclusions:** Our study provides a layer-by-layer-based co-culture platform to support the prolonged survival of primary IECs and their features, which is important for understanding IEC function in response to the gut microbiota.

## Introduction

The intestinal epithelium is composed of a single layer of intestinal epithelial cells (IECs) that restrict the entry of harmful substances and respond to extracellular stimuli [1]. These IECs are tightly bound together, forming a physical barrier and securing the contents of the intestinal lamina, which is crucial to enhancing the function and integrity of IECs [2]. As a front line of the intestinal system, IECs contribute to the maintenance of intestinal homeostasis and the symbiotic relationship between gut microbiota residing in the intestinal lumen and the host [3]. IECs can respond to the gut microbiota or their metabolites and produce mediators that further impact other host intestinal immune cells, including innate lymphoid cells, macrophages, dendritic cells, natural killer cells, B cells, and T cells [4]. Upon sensing gut microbiota, certain protective barrier responses are activated to guard against bacterial infiltration into the host tissue, such as mucus production, lysozyme secretion, and the growth and differentiation of IECs [3,5–7]. Previous studies have shown that treatment with lipopolysaccharide (LPS), an outer membrane component of gram-negative bacteria, down-regulated the expression of tight junction (TJ) genes in IECs including E-cadherin, Claudin-1, and zona occludens 1 (ZO1) [8–12].

Multiple IEC culture systems have been established to study the functions of IECs [13]. The colonic epithelial immortalized cell lines in vitro, such as Caco-2 and HT-29, are the most extensively used method for determining IEC function in response to stimuli [14,15]. Although these immortalized cell lines recapitulate some physiological features of the intestinal epithelium, their use is still limited as these cells are mostly generated by genetic modification and are obtained from nonspecific intestinal regions and contaminated with other cell types [16,17]. In addition, various experimental approaches have been proposed to demonstrate a culture system that includes primary IEC growth and cellular differentiation into specialized cell types [18,19]. For example, intestinal organoids were generated by culturing IECs derived from either intestinal crypt cells or intestinal stem cells in a 3-dimensional environment, supplemented with essential growth and expansion factors, including R-spondin-1, epidermal growth factor (EGF), Noggin, and laminin-rich Matrigel [20]. This culture method showed small intestinal epithelium trademarks along with crypt-villus morphological organization, suggesting that it is optimal for studying the mechanism of IEC differentiation and self-renewal processes in both mouse and human colonic epithelia [21]. Although the intestinal organoid culture was developed as a multiplex screening platform, the need for special techniques for the co-culture of IECs with other cell types or intestinal microbiota limits this development [22,23]. Furthermore, substantial improvements have been identified in biomimetic gut-on-a-chip compared to conventional in vitro culture or organoid culture systems [24,25]. Despite being a powerful tool for studying the impact on IECs, setting up this method is complicated and requires numerous special techniques, indicating considerable limitations of existing methods. This has necessitated the development of alternative culture methods for primary IECs to facilitate functional IEC studies.

A previous report described a new co-culture method for generating 2 layers of cell assembly through cell surface engineering [26]. Through the mild reduction of disulfide bonds, the cell surface was fabricated into a reactive state by exposing the free active thiol at the outer membrane of the cells [27]. These thiol-rich cell surfaces could be further chemically grafted with maleimide chondroitin sulfate (MCS) via the thiol–maleimide coupling reaction, thereby generating a negatively charged cell surface [28]. A reversible cell-layering platform was assembled by adding a positively charged chitosan film to the MCS-grafted cells and the intermediate porous chitosan thin films were formed via ionic cross-linking between anionic polymer MCS and cationic polymer chitosan, allowing for direct cell–cell contacts between co-cultured cells [26,28]. Crosstalk between IECs and their niche, including extracellular matrix (ECM), fibroblasts, and growth factors, is required to properly maintain the growth and function of IECs [20,29,30]. Notably, intestinal fibroblasts, located in proximity to IECs, provide crucial chemical and physical signals, including Wnt and Notch, to regulate IEC functions such as proliferation, localization, migration, and differentiation for the maintenance of barrier function [29,31].

Therefore, in this study, we adapted the previously reported reversible layer-by-layer (LBL) method mediated by chitosan films to layer primary IECs with fibroblasts as a novel co-culture platform. We hypothesized that a highly porous intermediate chitosan film would allow for direct intercellular interactions between fibroblasts and IECs, thus supporting the survival and function of IECs following the native intestinal niche. This study highlights a feasible and easy-to-use method for constructing a long-term culture platform for primary mouse IECs that allows for the presence of mature IECs, prolonged cell survival, and response to external stimuli.

## Materials and Methods

### Isolation of primary IECs from both the small intestine and colon

All animal experimental procedures were conducted in accordance with the protocol for animal handling and ethical standards approved by the Institutional Animal Care and Use Committee at Soonchunhyang University (Animal protocol number: SCH17-0003). Eight-week-old C57BL/6N male mice were purchased from ORIENT Bio Korea (Seoul, Republic of Korea) and placed in a specific pathogen-free animal facility at the Soonchunhyang Institute of Medi-bio Science. Figure S1 shows a schematic illustration of the primary IEC isolation. Briefly, to isolate primary IECs from 8-week-old C57BL/6N male mice, the small intestine and colon were collected in tubes containing ice-cold phosphate buffered saline (PBS, cat# 21031, Corning, NY, USA). After fat removal, the tissue was opened longitudinally, cut into 0.5-cm pieces, and washed with ice-cold PBS until the supernatant was clear. Small pieces of tissue were transferred into tubes containing 20 ml of 5 mM ethylenediaminetetraacetic acid (EDTA, cat# 03690, Sigma-Aldrich, St. Louis, MO, USA) and 5 mM dithiothreitol (cat# 43819, Sigma-Aldrich, St. Louis, MO, USA) in ice-cold PBS and then shaken at 150 rounds per minute (rpm) at 4 °C for 20 min. Tissues were then transferred to a tube containing 5 ml of 30 mM EDTA in PBS and shaken at 200 rpm and at 37 °C for 10 min. The digested solution was passed through a 70-μm strainer and centrifuged at 1,000 × *g* at 4 °C for 10 min. The IEC pellet was then washed with PBS (with calcium and magnesium, cat# 21030, Corning, NY, USA) containing 10% fetal bovine serum (FBS, cat# 26140079, Thermo Fisher Scientific, Waltham, MO, USA). After centrifugation, the IEC pellet was resuspended with complete growth media; RPMI (cat# 10404, Corning, NY, USA) containing 10% FBS, 1% penicillin–streptomycin (cat# 30002, Corning, NY, USA), 1% MEM nonessential amino acids (cat# 25025, Corning, NY), and 0.1% β-mercaptoethanol (cat# 21985023, Thermo Fisher Scientific, Waltham, MO, USA). The isolated number of IECs per mouse using this method ranged from approximately 1 × 10^6^ to 3 × 10^6^ cells for the small intestine and 5 × 10^5^ to 1 × 10^6^ cells for the colon. The cell viability of freshly isolated IECs was approximately 70% to 80% for both small intestine and colon tissues, calculated based on the ratio of live cell numbers to total cell numbers using trypan blue staining.

### TCEP treatment and quantitative evaluation of a free thiol on cell surface

To create LBL constructs, human dermal fibroblasts, purchased from Daewoong Pharmaceutical Company, were used as feeders. These fibroblasts were seeded onto tissue culture-treated glass bottom dish, allowed to attach, and then treated with Tris(2-carboxyethyl)phosphine (TCEP). In contrast, freshly isolated IECs (1 × 10^6^ cells) were collected in 15-ml tubes by centrifugation and resuspended in 1 ml of PBS containing TCEP. Both dermal fibroblasts and IECs were incubated with varying concentrations (0, 0.25, 0.5, or 1 mM) of TCEP diluted from 100 mM of TCEP stock dissolved in PBS (cat# C4706, Sigma-Aldrich, MO, USA) and then incubated with TCEP with varying concentrations at room temperature (22 °C) for 5 min. After TCEP treatment, cells were washed with PBS to remove TCEP residues. For the quantification of the amount of free thiols on cell surface, Ellman’s assay was performed using 10 mM of 5,5′-dithio-bis-(2-nitrobenzoic acid) (cat# 22582, Thermo Fisher Scientific, Waltham, MO, USA) by following the manufacturer’s instructions and L-cysteine (cat# C7352, Sigma-Aldrich, St. Louis, MO, USA) was used to establish the standard curve (Fig. S2). For Ellman’s assay, a working solution was prepared by diluting 50-fold of the stock solution and mixing it with the samples. Then, the mixture was incubated for 15 min at room temperature and absorbance was measured at 412 nm using a microplate reader (BioTek, Winooski, VT, USA). The optical density was calculated as a relative fold change.

### MTT assay

To assess cell viability and cytotoxicity of TCEP, cell viability was quantified by MTT assay. Prior to the assay a 12 mM stock solution of 3-(4,5-dimethylthiazol-2-yl)-2,5-diphenyltetrazolium bromide (MTT, cat# M6494, Invitrogen, Carlsbad, CA, USA) was prepared and diluted to 1.2 mM working solution using a fresh growth medium. Confluent fibroblasts adhered on the 48-well plate and suspension of IECs were treated with varying concentrations of TCEP and incubated with MTT working solution for 4 h at 37 °C. Relative absorbance differences were measured at 540 nm using a microplate reader (BioTek, Winooski, VT, USA).

### Visualization of free thiols on the cell surface

To visualize the presence of free thiols on the IEC surface, suspended epithelial cells and adherent human dermal fibroblasts cultured on tissue culture plates were treated with varying TCEP concentrations (0, 0.25, 0.5, or 1 mM TCEP). Then, these cells were incubated with Alexa Fluor® 488 C_5_ maleimide (working concentration of 3 μg/ml diluted in PBS, cat# A10254, Thermo Fisher Scientific, Waltham, MO, USA) for 10 min at room temperature. After treatment, cells were washed with PBS and imaged using a confocal microscope (LSM 710; Carl Zeiss, Oberkochen, Germany) at the Soonchunhyang Biomedical Research Core Facility of Korea Basic Science Institute (KBSI).

### Synthesis of maleimide-conjugated chondroitin sulfate

To incorporate the negatively charged chondroitin sulfate to the cell surface, maleimide-conjugated chondroitin sulfate (MCS) was synthesized using the modified version of a previously reported method [28]. Briefly, 2 g of chondroitin sulfate sodium salt (CS, cat# C0335, Tokyo Chemical Industry, Tokyo, Japan) was dissolved in 40 ml of PBS with vigorous magnetic stirring. Next, 286 mg of *N*-[3-(dimethylamino)propyl]-*N*′-ethylcarbodiimide hydrochloride (EDC, cat# 39391, Sigma-Aldrich, St. Louis, MO, USA) and 328 mg of *N*-hydroxysulfosuccinimide sodium salt (NHS, cat# 56485, Sigma-Aldrich, St. Louis, MO, USA) were dissolved in 10 ml of PBS. After the CS was completely dissolved, the mixture containing EDC and NHS was added to the CS solution and stirred for 20 min at room temperature. Then, 250 mg of *N*-(2-aminoethyl)maleimide trifluoroacetate salt (maleimide, cat# 56951-1G-F, Sigma-Aldrich, St. Louis, MO, USA) was dissolved in 10 ml of PBS, added to the mixture, and incubated for 5 h with gentle stirring at room temperature. Afterward, the solution was dialyzed with a dialysis membrane (cat#132676, Spectrum Laboratories, CA, USA) against distilled water for 4 days (distilled water was renewed every 12 h) at room temperature and freeze-dried until future use.

### MCS grafting on cell surface and development of a chitosan thin-film-based LBL co-culture platform

To induce MCS grafting on the surface of fibroblasts and IECs, these cells were treated with 1 mM TCEP for 5 min and then reacted with 10 mM MCS for 30 min at 37 °C. For feeder cells (fibroblasts), the solution was gently removed and 266 μl of Dulbecco's Modified Eagle Medium (DMEM) was added to the fibroblast layer, followed by the addition of 134 μl of 1.5 mg/ml chitosan (cat# 448869, Sigma-Aldrich, St. Louis, MO, USA) in a 1% acetic acid (cat# 1005-4105, Daejung Chemicals, Siheung, South Korea) solution. After homogeneously spreading solution to the cell surface, the sample was incubated for 45 min at 37 °C, and the opaque layer of a chitosan thin film was formed through electrostatic complexation. Then, MCS-grafted IECs were dispersed in a 2:1 mixture of 2 types of Matrigel, Matrigel® Basement Membrane Matrix (cat# 354234, Corning, NY, USA) and Geltrex™ (cat# A1413302, Thermo Fisher Scientific, Waltham, MO, USA). The mixture of cells and Matrigel was then seeded on top of chitosan film to generate layer-to-layer-based co-culture constructs and incubated for 2 h at 4 °C and consequently settle the floating IECs on the chitosan surface. Then, the construct was incubated at 37 °C for an additional 30 min to allow for the solidification of the IEC–Matrigel mixture. The cells were cultured at a 1:1 ratio of complete growth media and Intesticult^TM^ Mouse Basal Medium (cat# 06005, StemCell Technologies, Cambridge, MA, USA). To assess the response of IECs to bacteria-derived molecules without inducing cell death, the co-culture construct was cultured in the presence of LPS from *Escherichia coli 0111:B4* (cat# tlrl-3pelps, InvivoGen, San Diego, CA, USA) at a concentration of 1 or 10 μg/ml in the culture media, as previously reported [32].

### CellTracker dye transfer assay

Prior to generation of the thin film chitosan-based fibroblast-IEC co-culture constructs, cells were incubated in serum-free medium containing CellTracker Red CMTPX (5 μM; cat# C34552, Thermo Fisher Scientific, ON, Canada) for IECs and CellTracker Violet BMQC (2 μM; Thermo Fisher Scientific, ON, Canada) for fibroblasts for 45 min at 37 °C. Then, the cells were gently washed once with PBS to remove excess dye, prepared for TCEP treatment, MCS grafting, and chitosan-based LBL constructs, and cultured for 72 h with complete growth media at 37 °C and 5% CO_2_ level. Dye transfer and Z-stack images of chitosan thin-film-based co-culture of fibroblasts and IECs were captured using a confocal microscope (LSM 710; Carl Zeiss, Oberkochen, Germany) at the Soonchunhyang Biomedical Research Core Facility of KBSI. The efficiency of dye transfer was determined using ImageJ (NIH, USA) by calculating the percentage of cells positive for transferred fluorescence (from top to bottom or from bottom to top) divided by the total cell number. Images were taken for at least biological triplicates for the quantification.

### Live/dead assay

Cell viability was visualized by staining with a Live/Dead viability/cytotoxicity kit (cat# L-3224, Thermo Fisher Scientific, Waltham, MO, USA). The kit contained calcein-AM and ethidium homodimer-1 (EthD-1) for the staining of live and dead cells, respectively. Cells were imaged using a 20× objective lens via confocal microscopy (LSM 710; Carl Zeiss, Oberkochen, Germany) at the Soonchunhyang Biomedical Research Core Facility of KBSI. The percentage of cellular viability was determined using ImageJ (NIH, USA) by counting the number of live cells stained with calcein-AM divided by the total number of cells, including calcein-AM-positive and EthD-1-positive cells. Images were taken for at least biological triplicates for the quantification.

### Immunofluorescence

After medium removal, the co-culture construct was fixed with 4% paraformaldehyde (cat# CNP015-0500, CELLNEST, Hanam, South Korea) and then permeabilized with 0.1% v/v tween-20 (cat# P1379-100ML, Sigma-Aldrich, MO, USA) and 0.2% v/v Triton X-100 (cat# TRX777.500, BioShop, ON, Canada) in PBS. The samples were then incubated with 5% bovine serum albumin (cat# ALB001.100, ON, Canada) for 1 h and primary antibodies anti-Villin (1:200; cat# sc-58897, Santa Cruz Biotechnology, Santa Cruz, CA, USA) and anti-E-cadherin (1:200; cat# 3195S, dilution factor, Cell Signaling Technology, Danvers, MA, USA) at 4 °C overnight. The cells were then incubated with secondary antibodies: Goat anti-Rabbit IgG, Alexa Flour 488 (cat# A11008, Invitrogen, Waltham, USA) and Goat anti-Mouse IgG, Alexa Flour 488 (cat# A11001, Invitrogen, Waltham, USA) at room temperature for 2 h. Nuclei were stained with DAPI (cat# H21492, Thermo Fisher Scientific, Waltham, MO, USA) for 1 h. Stained cells were imaged using a confocal microscope (LSM 710; Carl Zeiss, Oberkochen, Germany) at the Soonchunhyang Biomedical Research Core Facility of KBSI.

### RNA isolation and reverse transcription-quantitative polymerase chain reaction analysis

To avoid mixing the 2 populations of cells during the RNA isolation, the Matrigel layer containing IECs was gently delayered using a spatula, ensuring physical separation from fibroblasts on the bottom of the plate (Fig. S6), and transferred to microcentrifuge tubes. To recover IECs embedded in Matrigel for further analysis, the Matrigel layer was digested with a Cell Recovery Solution (cat# 354253, Corning, NY, USA) and then centrifuged at 1,000 × *g* at 4 °C for 10 min. The cell pellets were resuspended in TRIZOL reagent (cat# 15596018, Ambion, CA, USA) for RNA isolation. RNA was converted to cDNA using reverse transcription reagents (cat# KMM-101, TOYOBO, Japan), according to the manufacturer’s instructions. qPCR was performed using the SYBR Green Real time PCR Master Mix Kit (cat# 4472918, TOYOBO, Osaka, Japan) with the primers listed in Table S1. The reaction was performed using an ABI StepOne plus real-time PCR machine (Applied Biosystems QuantStudio 5, CA, USA) at the Soonchunhyang Biomedical Research Core Facility of the KBSI. All experiments were performed with at least 3 biological replicates for each group, and the expression levels of genes of interest were normalized against glyceraldehyde-3-phosphate dehydrogenase (GAPDH). The ΔCt values were determined as follows: Ct^target^ − Ct^GAPDH^, and relative fold changes were calculated using the 2^−ΔΔCt^ method [33].

### Statistical analysis

Statistical analysis was conducted using GraphPad software (PRISM 8 GraphPad, CA) with a one-way analysis of variance (ANOVA). Values are presented as mean ± standard deviation. Statistical significance was set at *P < 0.05, **P < 0.01, and ***P < 0.001. The data presented in each experiment represent 3 independent biological experiments.

## Results and Discussion

### Cell surface modification by mild reduction using TCEP

**Fig. 1.**
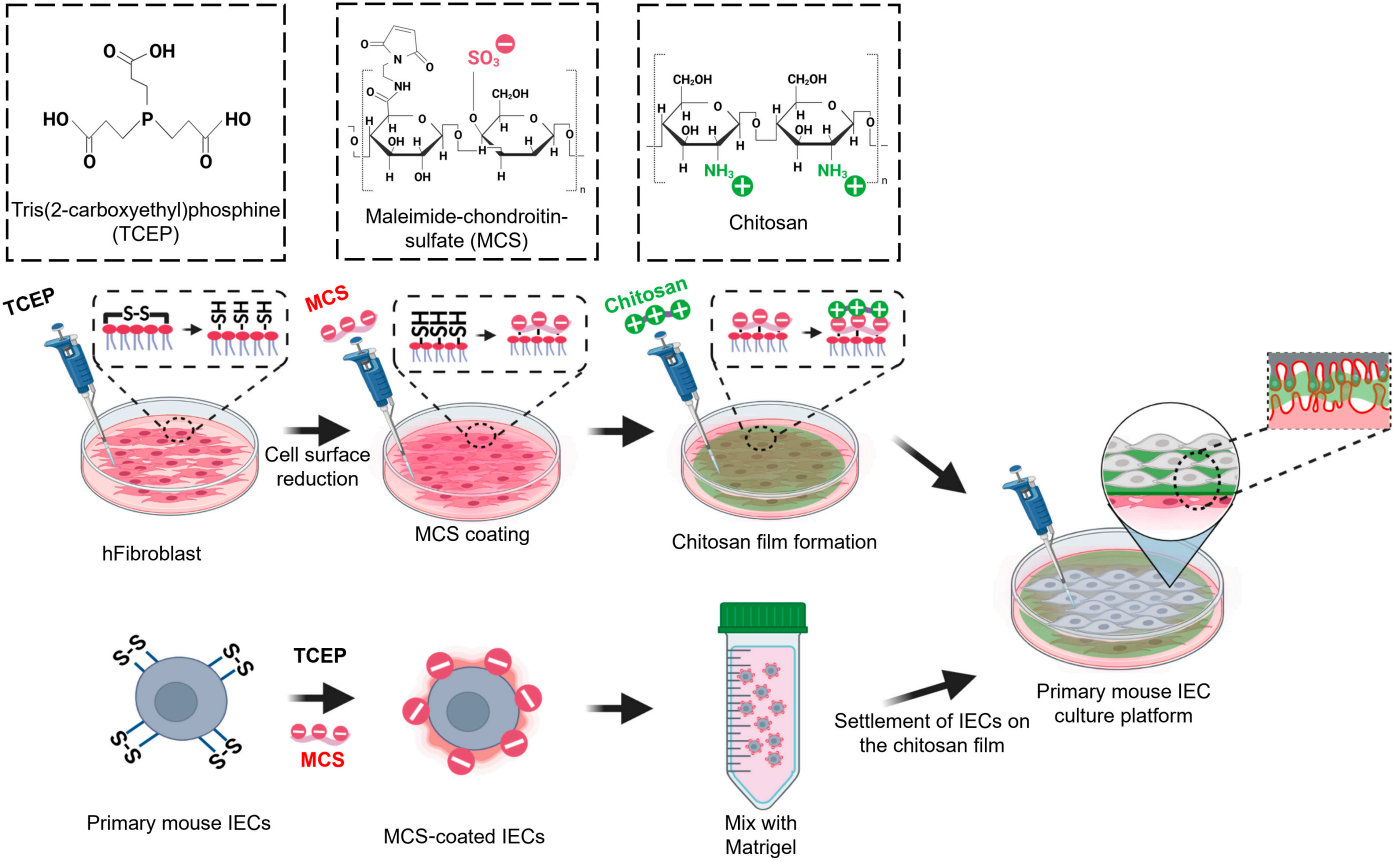
Schematic illustration of layer-by-layer (LBL) generation of the human fibroblast and IEC co-culture system as a biomimetic feature of the gut environment by the cell surface engineering method.

**Fig. 2.**
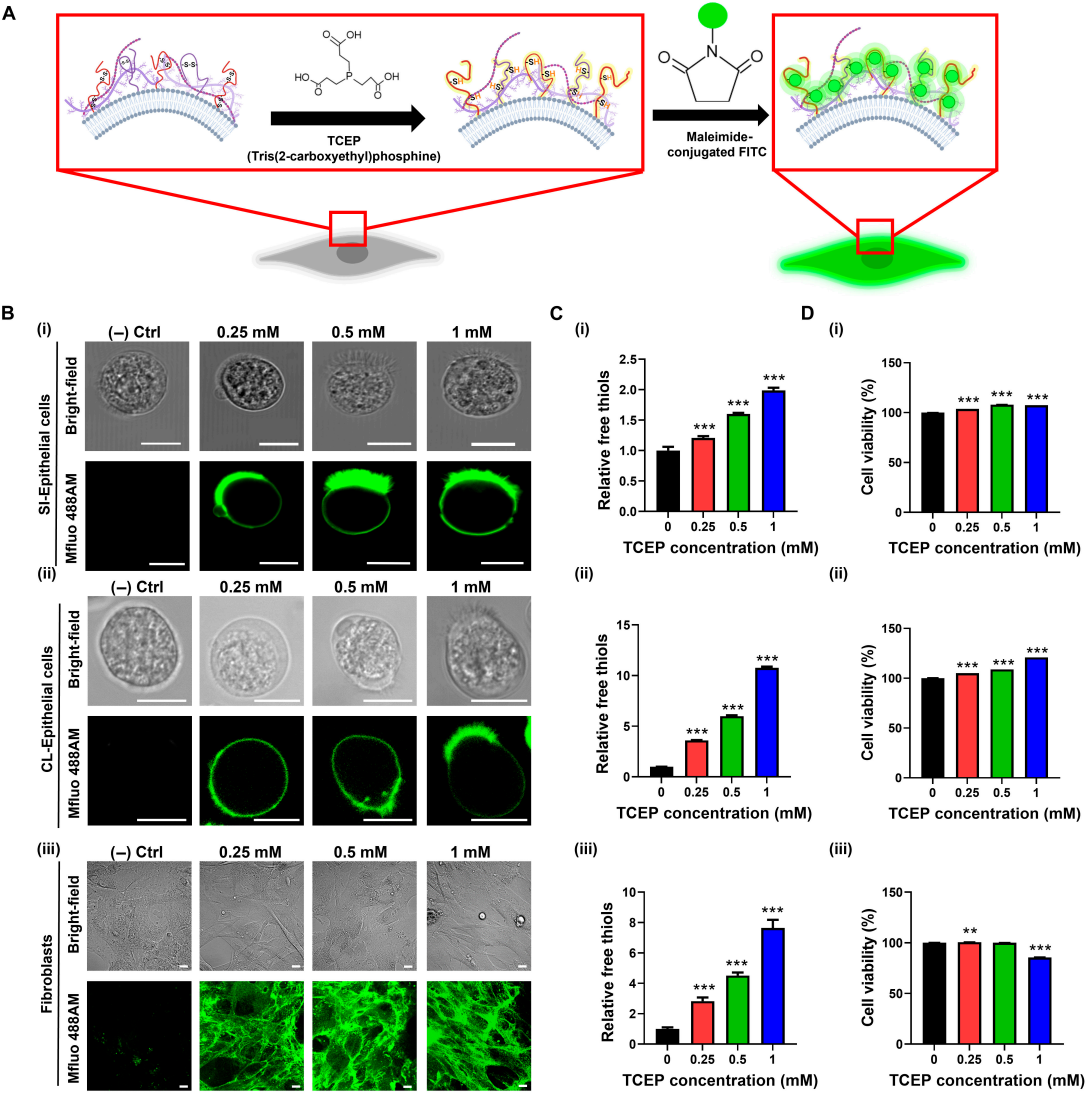
Surface engineering of human fibroblasts and mouse primary IECs via mild reduction. (A) Illustration of cell surface modification by mild reduction using TCEP; maleimide-conjugated green fluorescent dye (Mal-Alexa Fluor 488) was used to visualize thiol. (B) (Top) Confocal microscopic images of (i) small intestinal epithelial cells, (ii) colonic epithelial cells, and (iii) human fibroblasts. (Bottom) The presence of free thiol on cell surface was visualized with Mal-Alexa Fluor 488. Scale bar = 10 μm. (C) Ellman’s assay demonstrating the relative amount of free thiol produced by TCEP with varying concentrations (0 to 1 mM). (D) Viability of reduced cell compared to nontreated cell in different concentrations of TCEP. **P < 0.01 and ***P < 0.001 vs. the nontreated group (0 mM); *n* = 4 in panels C and D.

Using conventional IEC culture methods, the primary IECs could not be maintained ex vivo for more than 3 days and the IEC culture system failed to mimic the complex multicellular microenvironment within the gut niche in vivo [34]. Therefore, to develop a biomimetic cell layer for primary mouse IEC culture, we established a cell surface engineering method to co-culture fibroblasts with freshly isolated primary mouse IECs. This approach was adapted from our previously reported study [28] and the detailed process is illustrated in Fig. 1**.** Free active thiol groups of both fibroblasts and IECs are induced by the reduction of disulfide bonds using the mild reductant TCEP as a powerful reducing agent that can break disulfide bonds within and between proteins, exposing sulfhydryl groups or thiols on the cell surface [35,36]. To optimize the generation of free thiol groups on cell surface, we varied TCEP concentrations in the range of 0 to 1 mM. We exploited a fluorescent dye conjugated with a maleimide group (Mal-Alexa Fluor 488), which was used to visualize the active thiol groups on cell surface (Fig. 2A). The results showed that treatment with 1 mM TCEP showed a maximum green fluorescence signal on the surface membrane compared to other concentrations, indicating the presence of thiols on the surface of the target cells (Fig. 2B). In addition, thiol production on cell surface and cell viability upon TCEP treatment were assessed by using Ellman’s and MTT assays, respectively. The results showed that cells reduced with 1 mM TCEP demonstrated the highest thiol production while maintaining cell viability for both IECs and fibroblasts (Fig. 2C and D). Previously, it has been recognized that elevated concentrations of TCEP may have the potential to induce DNA strand breaks and elevate oxidative stress, ultimately leading to cellular apoptosis [37]. Therefore, we designated 1 mM TCEP as the optimal dose for breaking disulfide bonds without interfering with cell viability. This free thiol group at the cell interface can then be conjugated with maleimide groups because of its reactivity to cysteine or reactive thiol via a Michael-type addition reaction [38,39].

### LBL-based co-culture platform to mimic the interaction between fibroblasts and IECs in the native gut

The gastrointestinal tract comprises multiple cell types that support each other’s viability and function. For example, intestinal fibroblasts maintain epithelial stem cells and stimulate the proliferation, subsequent differentiation, and self-renewal of IECs [40]. In addition, they play a role in the maintenance of the intestinal stem cell niche, mediated by the production of essential growth factors [41,42]. Co-culture methods such as Transwell and conditioned medium showed the importance of fibroblasts as they provide mitogenic growth factors, including hepatocyte growth factor, and biochemical signals such as Wnt, which induce IEC proliferation in vivo or in vitro [40,43]. To exploit the beneficial effect of the interaction between fibroblasts and IECs, we used fibroblasts as feeder cells for co-culture with primary mouse IECs using the LBL platform (Fig. S3A). Previous studies were substantially limited as they failed to create direct contact between the 2 cell populations. Direct contact between fibroblasts and IECs provides abundant endogenous ECM and basement membranes, which are important for cell growth [44].

In this study, we used a porous thin film of positively charged chitosan to allow for direct contact between fibroblasts and IECs, both of which exhibit negatively charged cell surfaces due to the presence of negatively charged MCS. The z-stack confocal side-view images demonstrated the layer of IECs on top of fibroblasts, mediated by the chitosan layer forming the interface layer between IECs and fibroblast (Fig. S3B and C). Although we did not visualize the chitosan layer with fluorescence labeling in this study, we previously validated the formation of chitosan by utilizing fluorescein isothiocyanate (FITC)-conjugated chitosan to visualize the chitosan layer [26].

**Fig. 3.**
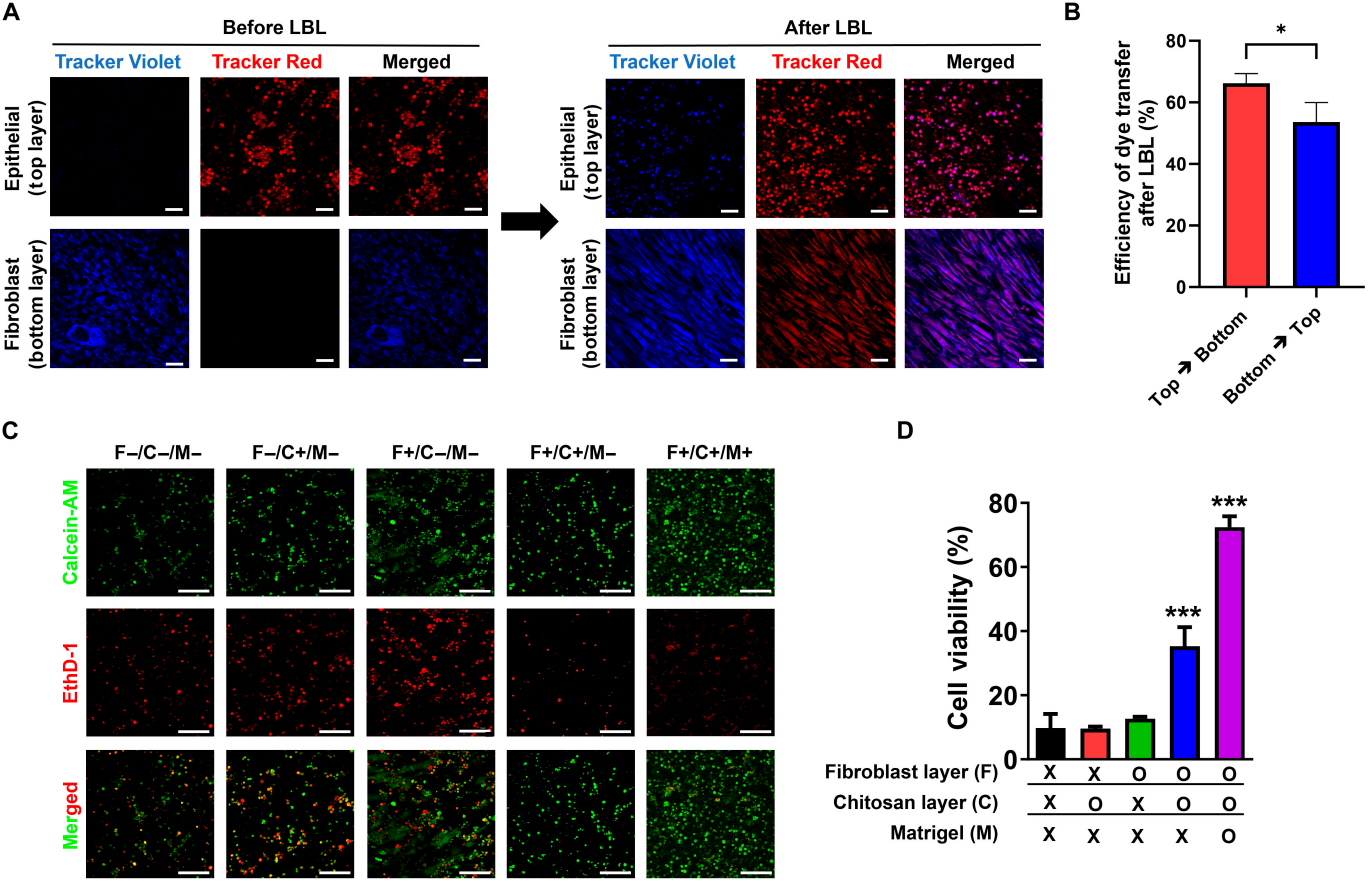
LBL system for co-culturing human fibroblasts and mouse primary IECs. (A) Fluorescent dye transfer between human fibroblasts (prelabeled with CellTracker Violet) and IECs (prelabeled with CellTracker Red) after 1 day of co-culture. Scale bar = 50 μm. (B) The efficiency of dye transfer was determined using ImageJ (NIH, USA) by calculating the percentage of cells positive for transferred fluorescence (from top to bottom or from bottom to top) divided by the total cell number. Images were taken for at least biological triplicates for the quantification. (C) Live/dead assay. Live cells were stained with calcein-AM (green) and dead cells (red) were stained with EthD-1. (D) Quantitative evaluation of IEC viability with varying cell culture conditions required for the LBL system. The presence of each cell culture condition (F: Fibroblast, C: Chitosan, M: Matrigel) was indicated by a plus sign (+) for presence or a minus sign (−) for absence. ***P < 0.001 vs. the nontreated group (0 mM); *n* = 3 except in F+/C+/M− *n* = 7. Scale bar = 100 μm.

To investigate whether the LBL platform allows and supports the cell-to-cell direct contact across chitosan, a fluorescent dye tracker assay was used to assess the molecular passaging between the cells in different layers. As shown in Fig. 3A, fibroblasts on the bottom layer were initially labeled with violet fluorescence, while IECs on the top layer were labeled with the red fluorescence, respectively. While the dye tracker is known to be impermeable and typically cannot be transferred to neighboring cells, it can still be transferrable through direct cellular contact between adjacent cells via gap junctions [45,46]. Gap junctions are intercellular channels formed between closely adjacent cells, allowing the transport of small molecules, those with a molecular weight of less than 1 kDa [47]. After employing the LBL method to co-culture IECs on top of fibroblasts, we observed the transfer of fluorescent dyes in both fibroblasts and IECs. Our results demonstrated the visualization of both violet and red fluorescent dyes, with molecular weights of 334.2 Da and 686.3 Da, respectively, in each layer (Fig. 3A and Fig. S4). The dye transfer efficiency from the top layer (IECs) to the bottom layer (fibroblasts) was approximately 66%, while the efficiency from the bottom layer (fibroblasts) to the top layer (IECs) was approximately 55% (Fig. 3B). These findings suggest that the chitosan thin film may function as a gap junction channel, enabling direct intercellular transfer—the process of transferring small molecules directly between the top and the bottom layers. Furthermore, previous studies have demonstrated molecular transfer between distinct cell layers through a direct co-cultured system [48,49]. Gap junctions, recognized as key components of epithelial junctions, play a crucial role in facilitating intercellular and intracellular interactions among epithelial cells. They contribute to communication and enable the passage of solutes and molecules between cells [50,51]. Proper cell communication through cell junctions is a key feature in regulating the growth, development, and tissue function of IECs [5,50,52]. Therefore, our results demonstrated that chitosan supports direct cell–cell communication between fibroblasts and IECs, similar to the native epithelial junction, allowing for the intercellular transfer of cellular components. Utilizing the LBL platform enables the close co-culture of primary IECs with fibroblasts, providing direct intercellular signals that support the growth of IECs.

### Culture of IECs using the LBL platform exhibits prolonged survival

Using the conventional 2-dimensional cell culture model, without feeder cells or ECM, cell viability determined by calcein-AM and EthD-1 staining showed that most primary IECs failed to maintain their viability within 24 h (Fig. S5). Therefore, we aimed to establish a long-term culture of primary IECs to improve the viability of IECs using our newly developed LBL platform. To identify the role of each condition, including feeders, chitosan thin film, and Matrigel utilized to support IEC growth in this model, the viability of IECs was assessed after 24 h of culture with varying culture conditions **(**Fig. 3B and C). The results showed that the viability of IECs was approximately 10% in the traditional culture (F−/C−/M− group), whereas the viability of IECs slightly increased to 13% in the presence of fibroblasts in the bottom layer, acting simply as a feeder. This result is consistent with previous studies showing that co-culturing IECs with fibroblasts could increase the survival rate of IECs and induce in vivo epithelial morphology in cultured IECs [44,53,54]. Moreover, viability was increased up to approximately 35% in the presence of both fibroblasts and chitosan layers, suggesting that direct contact between cells through chitosan in our LBL culture system could allow for intercellular transfer of the small molecules, which eventually supports the viability of IECs. In addition, the highest IEC viability was achieved when Matrigel was incorporated in the system, approximately 72%. Matrigel has been used in IEC culture systems because it allows for prolonged expansion and differentiation, which ease culturing [55]. In this study, Matrigel was used as an ECM supplement, which acts as a reservoir of growth factors that orchestrate the physiological function of IECs [56,57]. The components used in the culture of primary IECs by the LBL platform synergistically support IECs viability by providing growth factors from the ECM and other cells, similar to the natural gut environment.

**Fig. 4.**
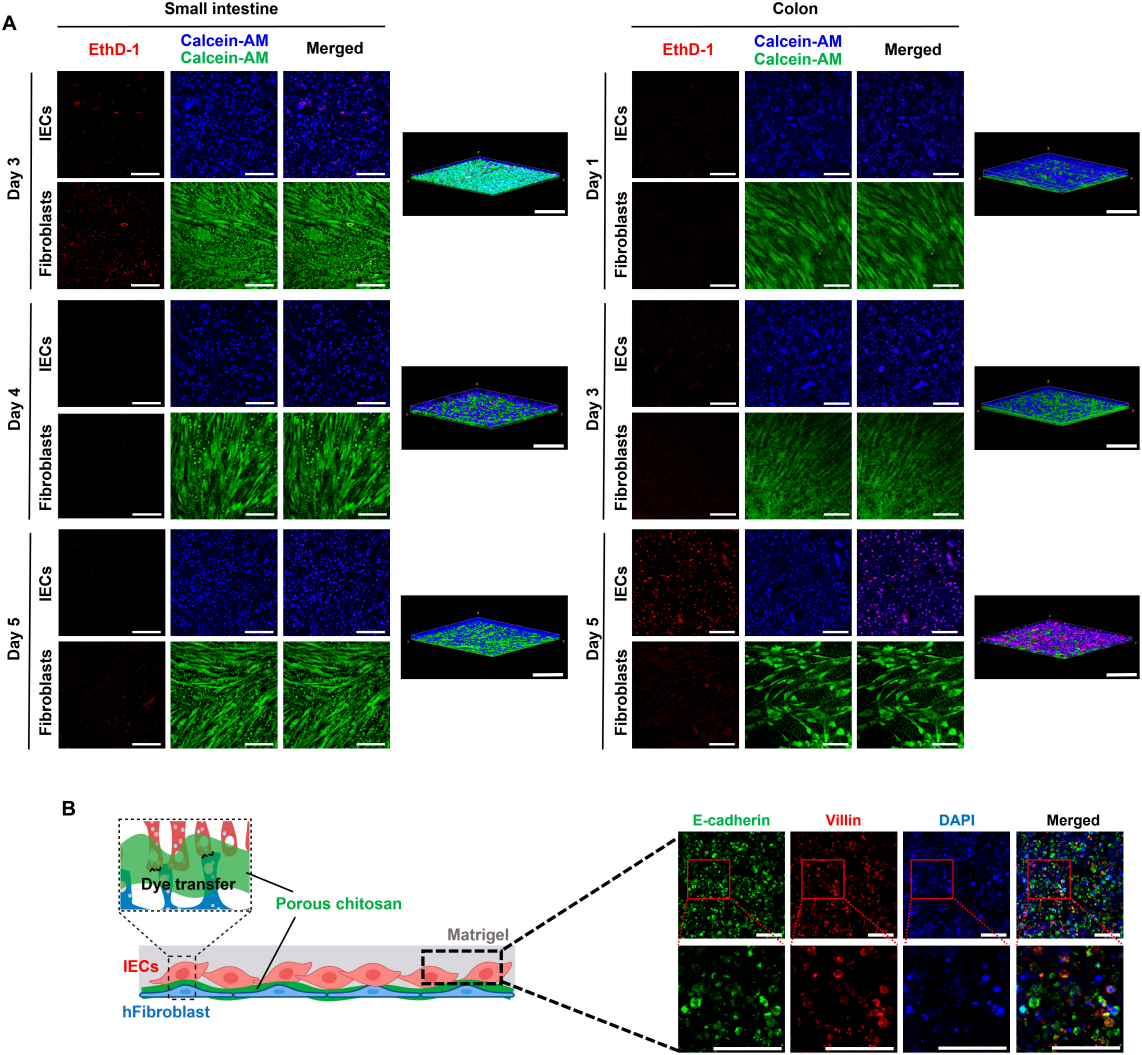
Viability and characteristic of cultured IECs by the LBL platform. (A) Live/dead assay. Live cells were stained with calcein-AM (green/blue) and dead cells (red) were stained with EthD-1. Viability of IECs cultured by this co-culture system. Scale bar = 100 μm. (B) The LBL system retained the IEC signature protein expression, villin, and E-cadherin. Scale bar = 50 μm.

We also examined whether the viability of IECs could be prolonged using the LBL culture method. Freshly isolated IECs were cultured using the LBL platform and cell viability was determined using cytotoxicity tests at different time points. The viability of small intestinal IECs was observed at 3, 4, and 5 days of culture, while that of colonic IECs was observed at 1, 3, and 5 days of culture (Fig. 4A and Fig. S6). The results showed that the viability of both small intestinal and colonic IECs could be prolonged for up to 5 days. In addition, the percentage of cell viability was calculated, and the results showed that the percentage of cell viability of small intestinal IECs was approximately 80% to 90%, while the percentage of cell viability of colonic IECs was approximately 70% to 80% after 5 days of culture. However, numerous EthD-1-positive cells representing dead cells were observed on day 5 of colonic IECs culture. In addition to the observation of IECs, fibroblasts were calcein-AM positive throughout the culture duration (up to 5 days), suggesting that the cell viability of both cells could be prolonged by at least 5 days using the LBL culture platform. In addition, this phenomenon is comparable to the in vivo condition, where a single layer of IECs self-renew every 4 to 5 days [58]. As the outermost barrier of the gut, IECs are dynamic and have a rapid renewal rate to maintain intestinal homeostasis [58–60]. Compared to previous studies, we demonstrated a more optimized platform for culturing primary IECs with prolonged cell viability. Maintaining cell anchorage to the ECM is the major challenge in culturing primary IECs in vitro. Cell anoikis leads to a high apoptotic rate, which is a hallmark of difficulties in primary IEC cultures [34]. Hofmann et al. also revealed the important role of cell–cell contacts in preventing anoikis of IECs. They found that even in the absence of cell–matrix anchorage, the initiation of the anchorage-dependent apoptotic caspase cascade in IECs can be inhibited by preserving the cell–cell contacts [61]. Therefore, through the LBL method, we improved this outcome using Matrigel as an ECM supplement to support cell–matrix interactions. Moreover, our platform provided direct cell–cell contacts between fibroblasts and IECs to allow for the transfer of growth factors and crosstalks between the cells, which may support the growth of IECs and prolong their viability [62]. Taken together, these results suggest that the LBL platform can serve as an alternative culture platform to prolong IEC survival. [61]

### Culture of IECs using LBL platform maintains their features and function in response to bacteria-derived molecules

Establishing a primary IEC culture model that can recapitulate key features of native IECs, such as TJs and the expression of differentiation markers, is important [63]. To determine whether the features of primary IECs were maintained when the primary IECs are cultured using the LBL platform, the expression of IEC signature proteins, including E-cadherin and Villin, was determined by immunofluorescent staining **(**Fig. 4B). E-cadherin is the core component of IEC integral membrane proteins, which are strongly and evenly expressed by IECs in both the small intestine and the colon [64,65]. Villin is an actin-regulating protein expressed in absorptive IECs and is generally used as a structural marker [66]. The results showed that green and red fluorescent signals representing E-cadherin and Villin, respectively, were observed in cultured IECs, suggesting that the key features of primary IECs are maintained by the LBL platform.

**Fig. 5.**
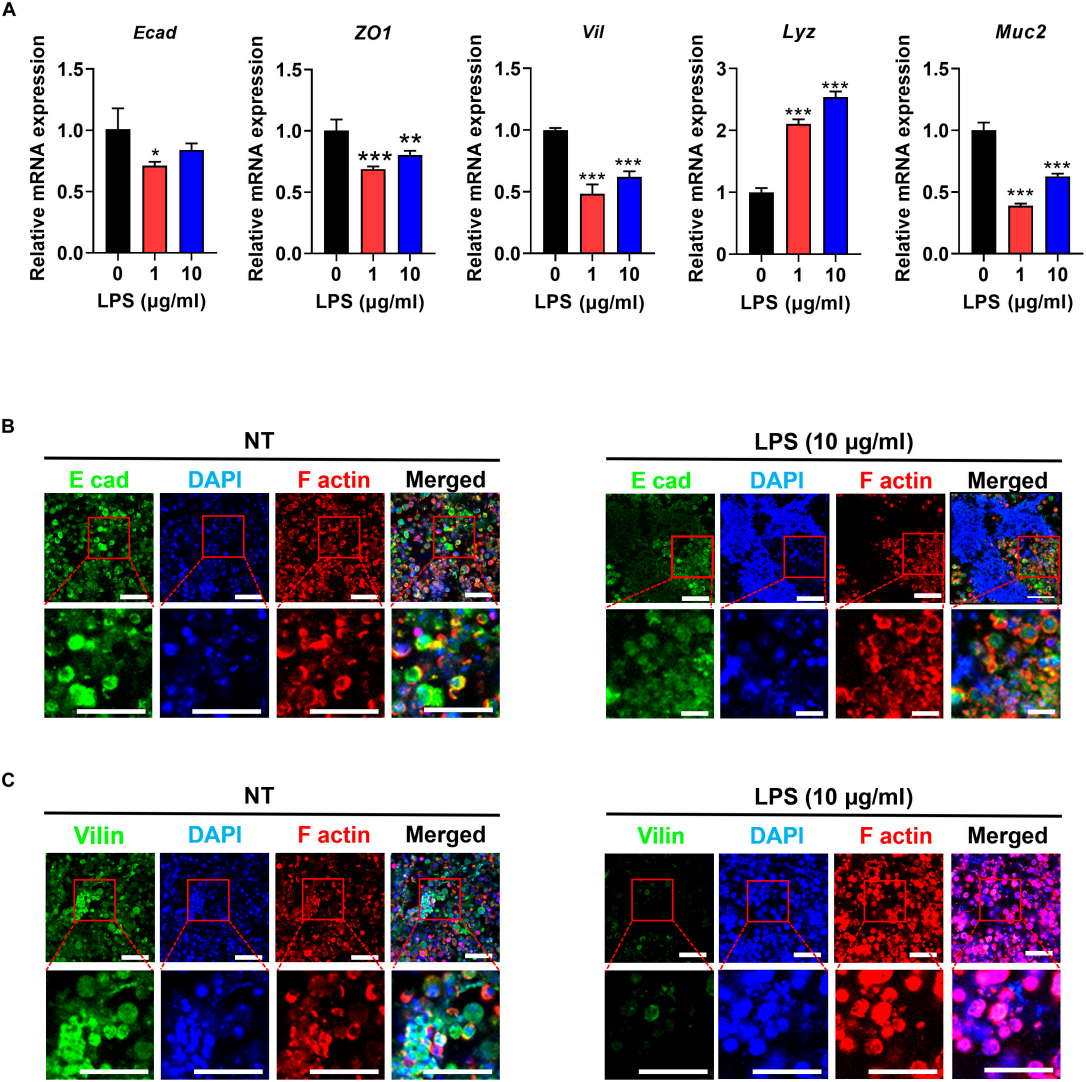
Response of cultured IECs to bacteria-derived molecules, lipopolysaccharide (LPS), in the LBL platform. (A) Relative gene expression of IECs cultured by the LBL system in the presence of LPS (1 or 10 μg/ml) compared with the nontreated group. *P < 0.05, **P < 0.01, ***P < 0.001; *n* = 3. Immunofluorescence staining of E-cadherin (B) and villin (C) on the layer of IECs in response to the 10 μg/ml LPS. “NT” indicates the nontreated group. Scale bar = 50 μm.

IECs are in constant contact with the microbiota in the intestinal tract. IECs can sense microbiota- and microbiota-derived signals to maintain intestinal homeostasis [67]. IEC culture systems have been extensively used to determine IEC function in response to stimuli, especially bacterial molecules existing in the gastrointestinal tract [13]. The response of IECs to bacteria-derived molecules was examined to determine the function of cultured IECs using this culture platform. In this study, a major component of the outer membrane of gram-negative bacteria, LPS, was used as a stimulus to determine the response of IECs. The co-culture construct was cultured in the absence or presence of LPS at concentrations of 1 or 10 μg/ml, and the expression levels of E-cadherin (*Ecad*), ZO1, villin (*Vil*), lysozyme (*Lyz*), and mucin 2 (*Muc2*) were quantified by qPCR **(**Fig. 5A). E-cadherin is an adherens junction that forms intercellular contacts between IECs. ZO1 is a peripheral membrane adapter protein that is crucial for the regulation and maintenance of TJ structures [68]. Villin is mainly produced in IECs that develop a brush border responsible for absorptive functions [66]. Lysozyme and mucin 2 are expressed by the Paneth and goblet cells, respectively. Previous studies in vitro and in vivo have shown that LPS treatment downregulated the mRNA expression and protein levels of TJs and villin in IECs [8,9,12]. Our results consistently showed that the expression levels of *E-cad*, *ZO1*, and *Vil* decreased in IECs cultured using this culture platform **(**Fig. 5A). In addition, the downregulated protein levels of E-cadherin and villin were confirmed through immunofluorescence staining (Fig. 5B and C). In addition, the expression level of *Muc2* decreased, whereas that of *Lyz* increased in the cultured IECs upon LPS treatment. This result is consistent with previous in vitro and in vivo studies, demonstrating that LPS stimulation reduces TJ expression in Caco-2 cells. Furthermore, the administration of LPS in mice has been shown to result in villus shortening and increased gut permeability [69,70]. Thus, these results suggest that primary IECs cultured by the LBL platform maintain their characteristics and exert their function in response to bacteria-derived molecules.

The intestinal tract is a complex organ that comprises multiple compartments, including the intestinal epithelium, stromal cells, gut microbiota, and the local immune system. Intestinal lumen contains a community of gut microbiota and crosstalk between gut microbiota and IECs has been reported [71,72]. IECs express receptors that identify luminal gut microbiota and microbial metabolites [73,74]. In addition, crosstalk between IECs and intestinal immune cells or immune cell mediators influences IEC differentiation and function [75,76]. Therefore, our primary IEC culture method can be integrated with a co-culture of IECs, gut microbiota, or immune cells. Moreover, a porous polymer thin film containing primary IECs can be constructed for further experiments. Overall, our LBL platform provides additional tools for studying IECs in a complex intestinal environment.

## Conclusion

By mimicking the interaction between fibroblasts and IECs, we successfully developed an alternative culture platform called the LBL platform. We modulated the cell surfaces of primary IECs and fibroblasts to generate cell-to-cell contact, which was mediated by highly porous polymer thin films. Using this platform, the cultured IECs showed prolonged cell survival and maintained IEC features and functions, at least in response to bacteria-derived molecules. This newly developed culture platform is a feasible technique for culturing primary IECs. Furthermore, it allows for the study of the IEC function in the complex intestinal microenvironment by enabling culturing with other cell types in the intestine.

## Data Availability

The analyzed datasets generated during the study are available from the corresponding authors on reasonable request.
