# Supplementary material for: Cell Surface Modification-Mediated Primary Intestinal Epithelial Cell Culture Platforms for Assessing Host–Microbiota Interactions
Source: Biomater Res. 2024 Jan 25;28:0004. doi: 10.34133/bmr.0004 (PMC10845607; doi:10.34133/bmr.0004)
Supplement: Supplementary 1 — Figs. S1 to S6 Table S1 [file bmr.0004.f1.zip › Supplementary Legends.docx]

**
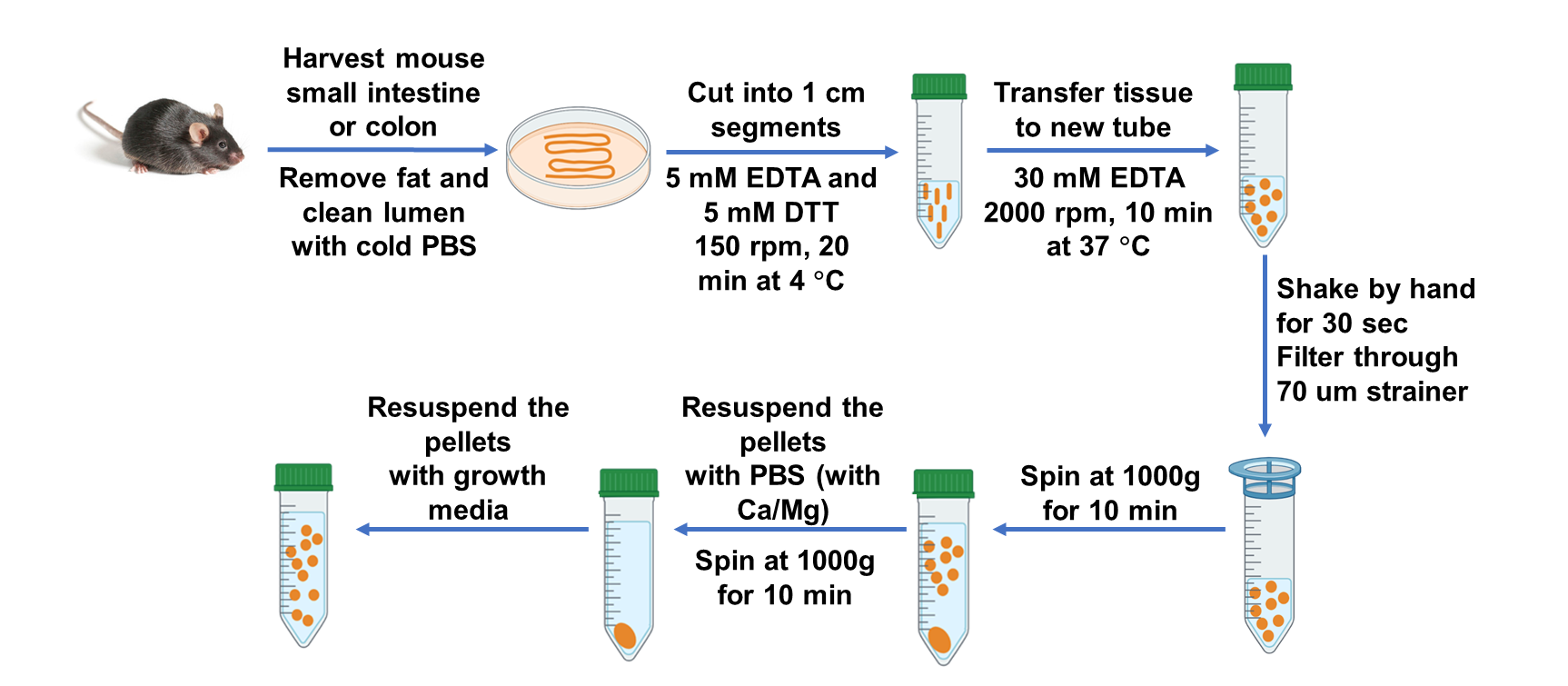
Supplementary Figure Legends**

**Supplementary Figure S1.** Isolation scheme of mouse primary intestinal epithelial cells (IECs). IECs were isolated from the small intestine and colon tissues.


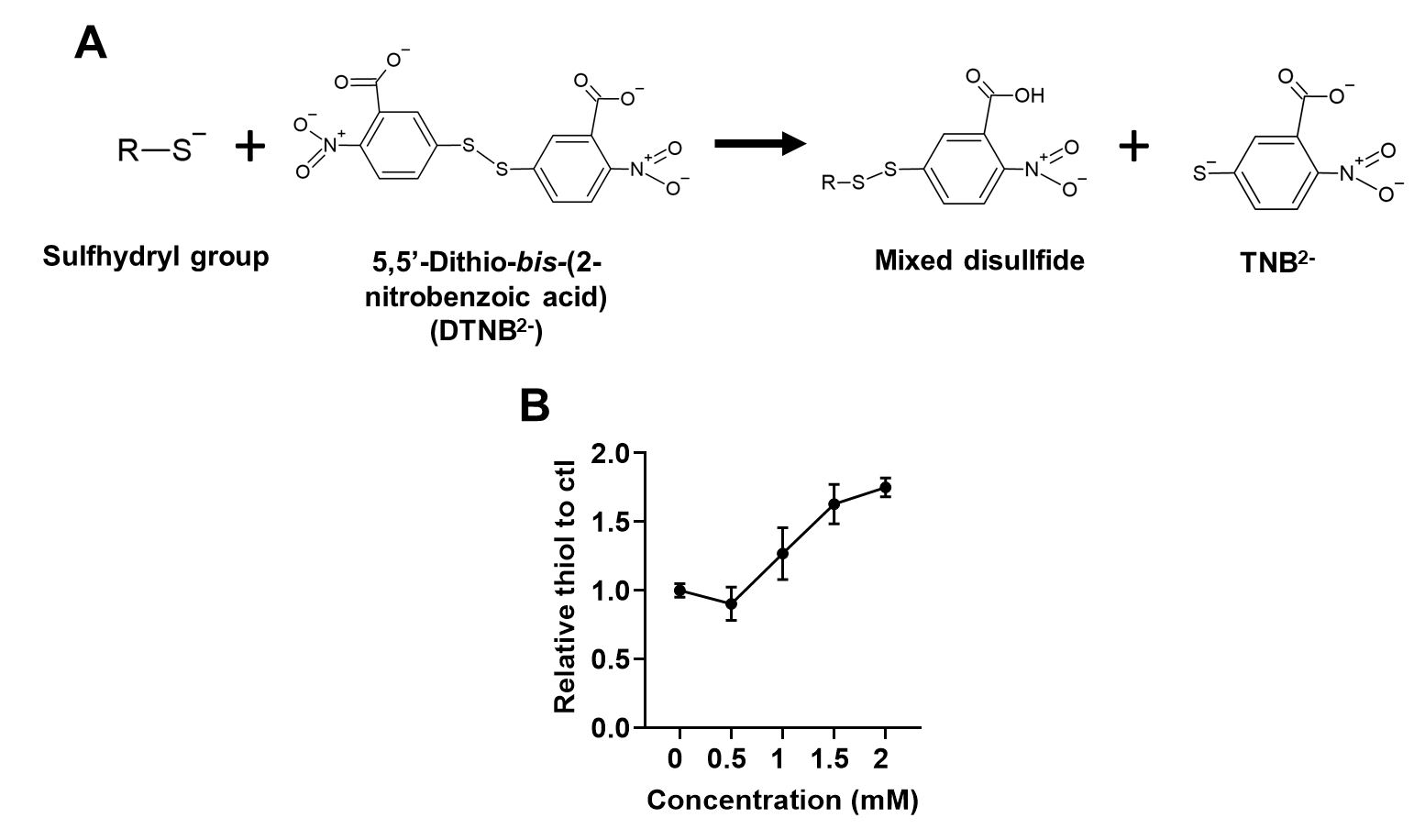


**Supplementary Figure S2.** Quantification of free sulfhydryl group by DTNB assay. (A) DTNB assay reduced the sulfhydryl group, leading to the production of yellow-species product, TNB^2-^, and (B) sulfhydryl quantification according to the cysteine standard. L-cysteine is diluted using reaction buffer as described in the protocol of the company.


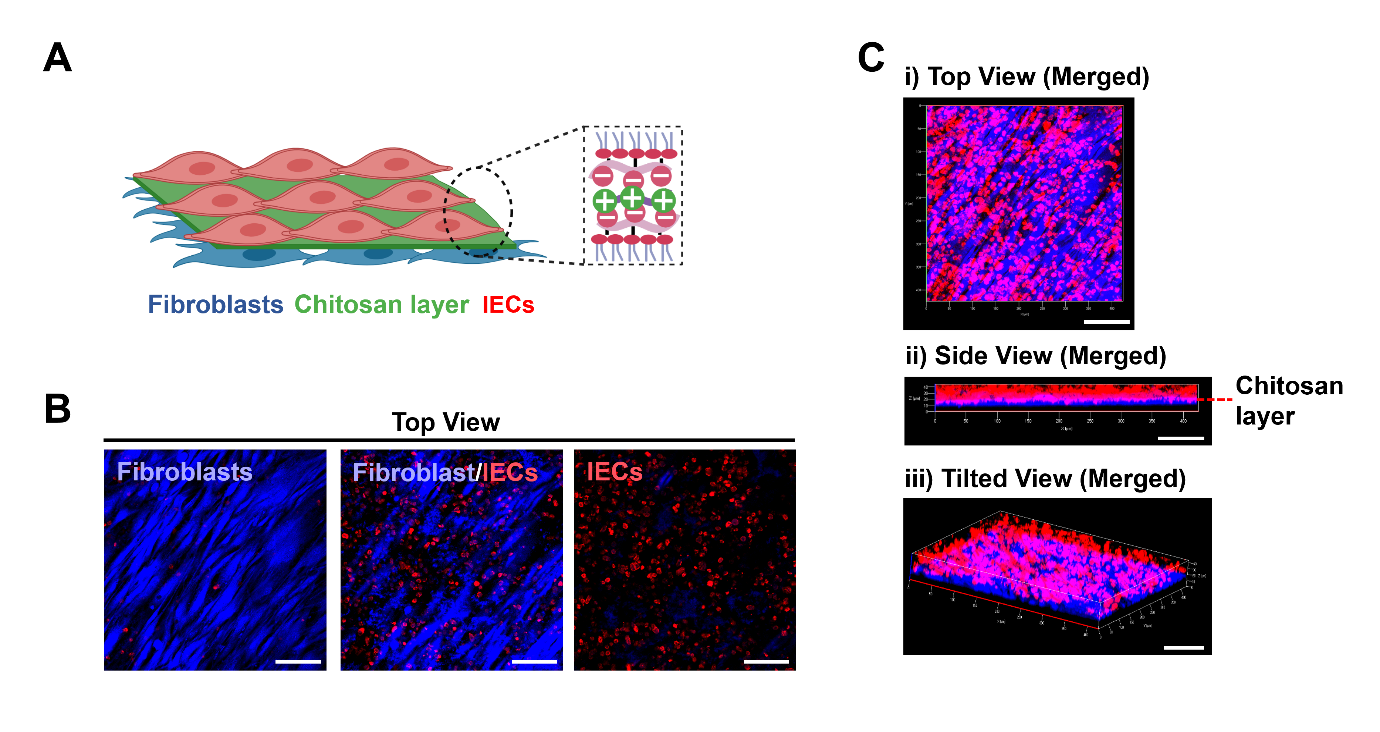


**Supplementary Figure S3.** Chitosan thin film-based co-culture system using layer-by-layer (LBL) cell stacking. (A) Drawing scheme of the LBL stacked cell. Chitosan formed as a barrier that separates the bottom cell (fibroblasts) and top cell (IECs). (B) Planar images of each layer show fibroblasts (bottom layer) emitting violet fluorescence, the chitosan layer serving as the interface layer (not labelled), and IECs (top layer) emitting red fluorescence. Scale bar = 100 µm. (C) 3D confocal images of the LBL from different angles: (i) top-view, (ii) side-view, and (iii) tilted view. Scale bar = 100 µm.


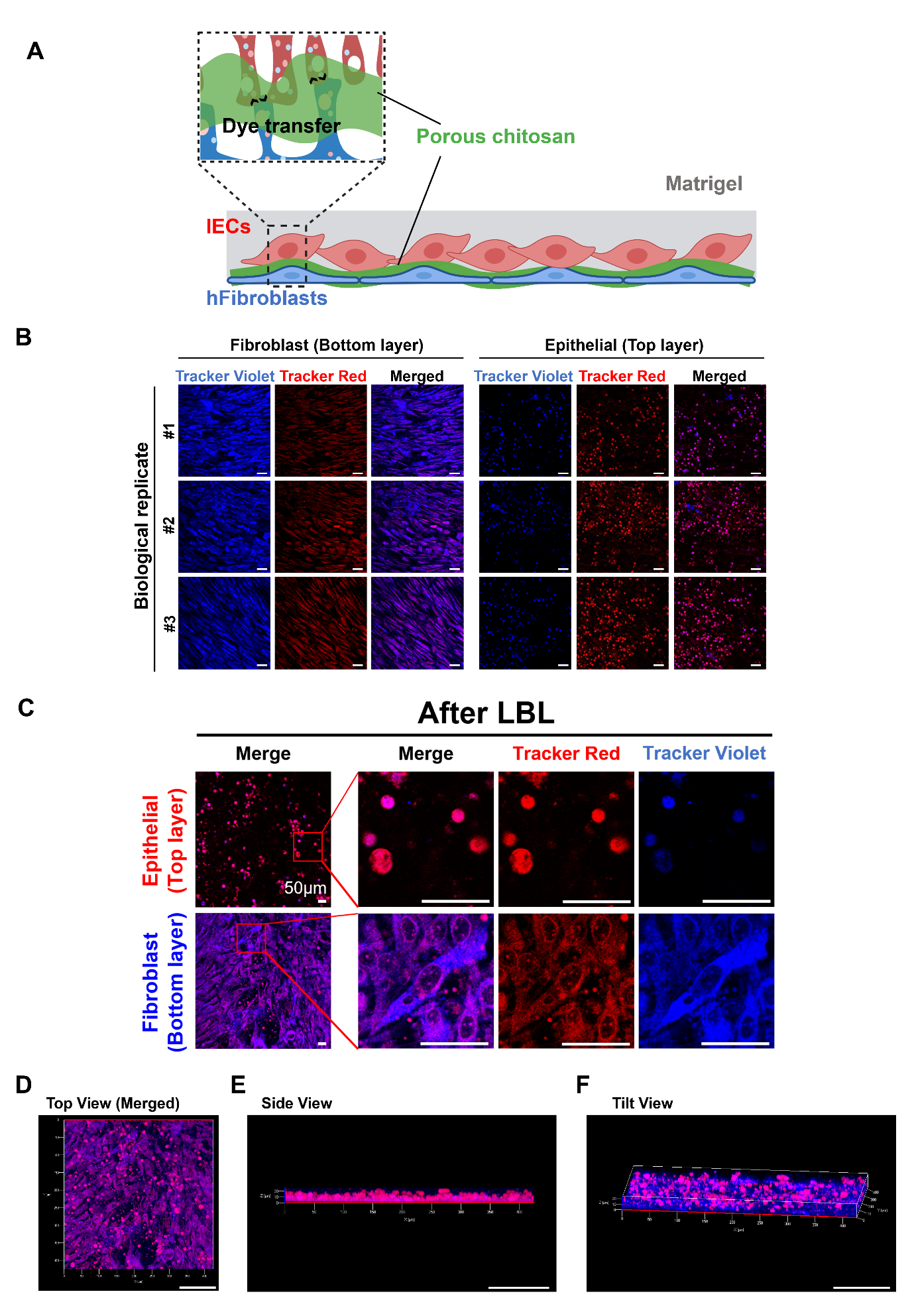


**Supplementary Figure S4.** Confocal images of the dye transfer between fibroblasts and IECs via chitosan layer in LBL system. (A) Schematics of layer-by-layer constructs using human fibroblasts (bottom layer), mouse primary IECs (top layer), and chitosan layer (middle layer). Prior to the layer-by-layer (LBL) process, fibroblasts labelled with violet fluorescence, while IECs labelled with red fluorescence. (B) Co-localization between the red and violet fluorescence of each layer after LBL was observed in different biological replicates. Fibroblasts (bottom layer) receive the red fluorescence from the top layer (top 🡪 bottom), while IECs receive violet, fluorescence from the fibroblasts (bottom 🡪 top). Scale bar = 100 µm. (C) Co-localization between the red and violet fluorescence of each layer after LBL was observed at high magnification. Scale bar = 50 µm. 3D confocal images of the LBL from different angles: (D) top-view, (E) side-view, and (F) tilted view. Scale bar = 100 µm.


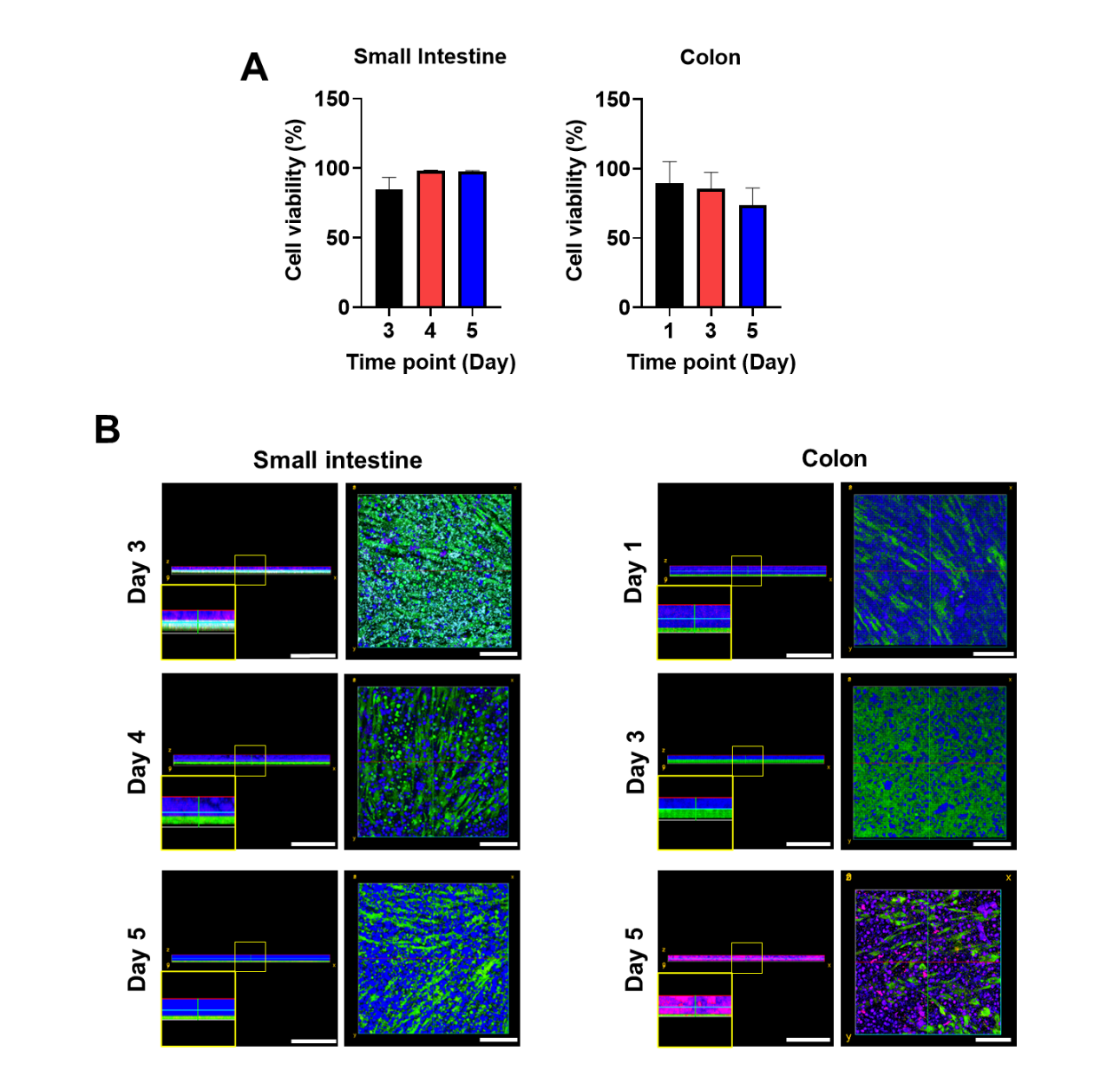


**Supplementary Figure S5.** Cell viability by layer-by-layer system. (A) Viability quantification of epithelial cell (IECs). (B) Side-view and top-view images of live (green) and dead (red) staining of small intestine and colon cells with layer-by-layer system. Scale bar = 100 µm.


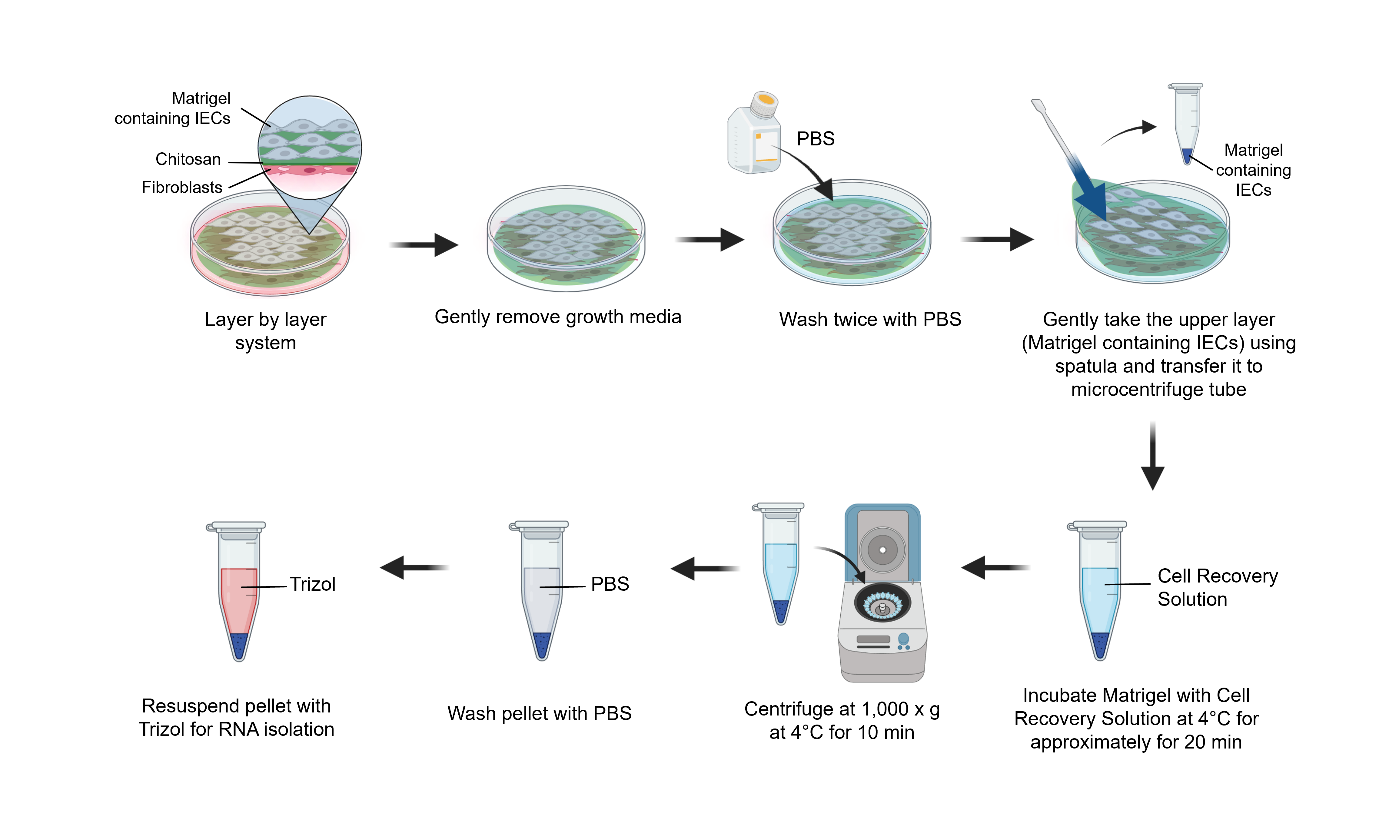


**Supplementary Figure S6.** Schematic representation of the delayering and isolation process of IECs from the layer-by-layer system. IECs can be easily harvested by gently detachment using a spatula and embedded IECs within Matrigel can be separated by Cell Recovery Solution (Matrigel-degrading solution).

| **Primers** | **Sequences (5’ – 3’)** |
| --- | --- |
| *Gapdh*_Forward | TTG ATG GCA ACA ATC TCC AC |
| *Gapdh*_Reverse | CGT CCC GTA GAC AAA ATG GT |
| *Zo1*_Forward | GGA GCT ACG CTT GCC ACA CT |
| *Zo1*_Reverse | GGT CAA TCA GGA CAG AAA CAC AGT |
| *Ecad*_Forward | ACC ACT GCC CTC GTA ATC GAA |
| *Ecad*_Reverse | CGT CCT GCC AAT CCT GAT GAA |
| *Muc2*_Forward | ATG CCC ACC TCC TCA AAG AC |
| *Muc2*_Reverse | GTA GTT TCC GTT GGA ACA GTG AA |
| *Lyz*_Forward | GAG ACC GAA GCA CCG ACT ATG |
| *Lyz*_Reverse | CGG TTT TGA CAT TGT GTT CGC |
| *Vil*_Forward | GAC GTT TTC ACT GCC AAT ACC A |
| *Vil*_Reverse | CCC AAG GCC CTA GTG AAG TCT T |

**Supplementary Table S1.** Lists of primers for quantitative PCR.
