# Supplementary figures and images for: Cell Surface Modification-Mediated Primary Intestinal Epithelial Cell Culture Platforms for Assessing Host–Microbiota Interactions
Source: Biomater Res. 2024 Jan 25;28:0004. doi: 10.34133/bmr.0004 (PMC10845607; doi:10.34133/bmr.0004)

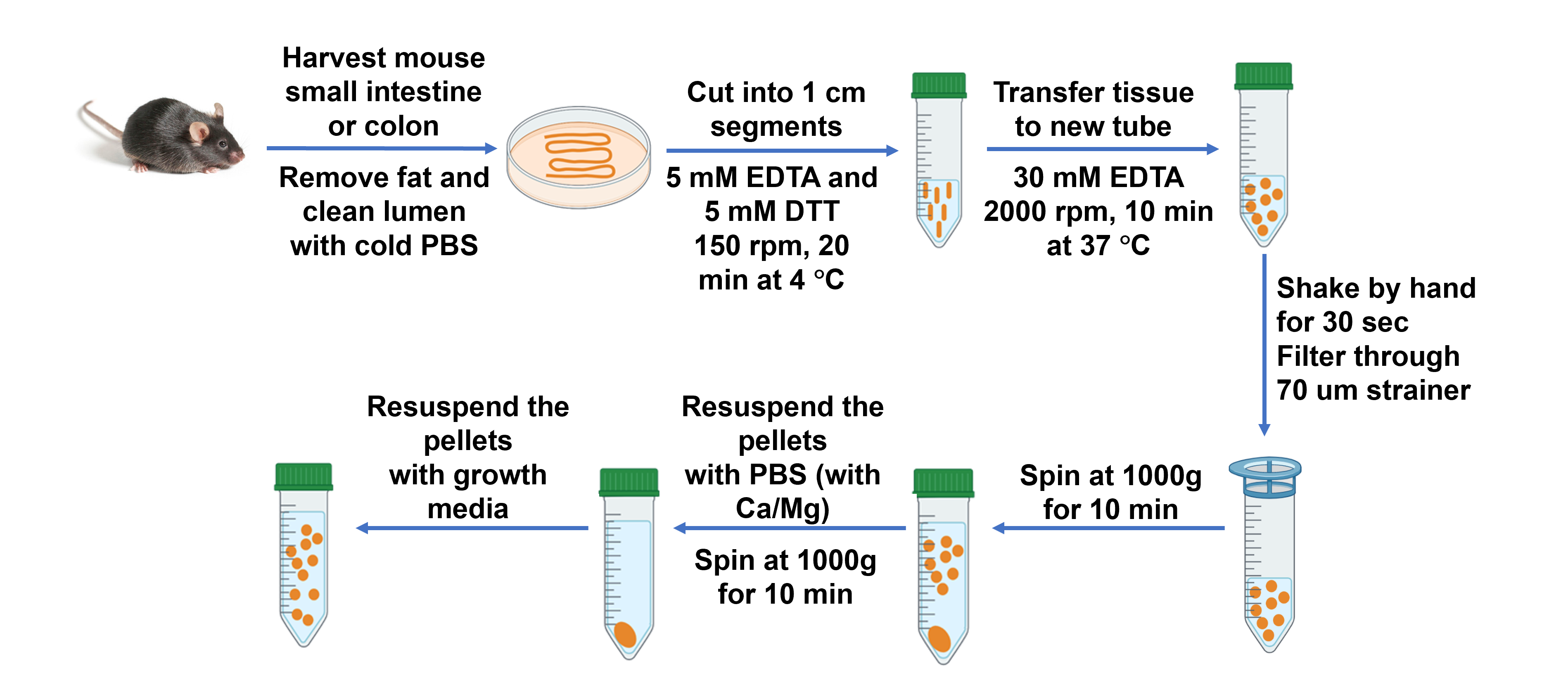

Supplement: Supplementary 1 — Figs. S1 to S6 Table S1 [file bmr.0004.f1.zip › Supplementary Figure S1.tif]

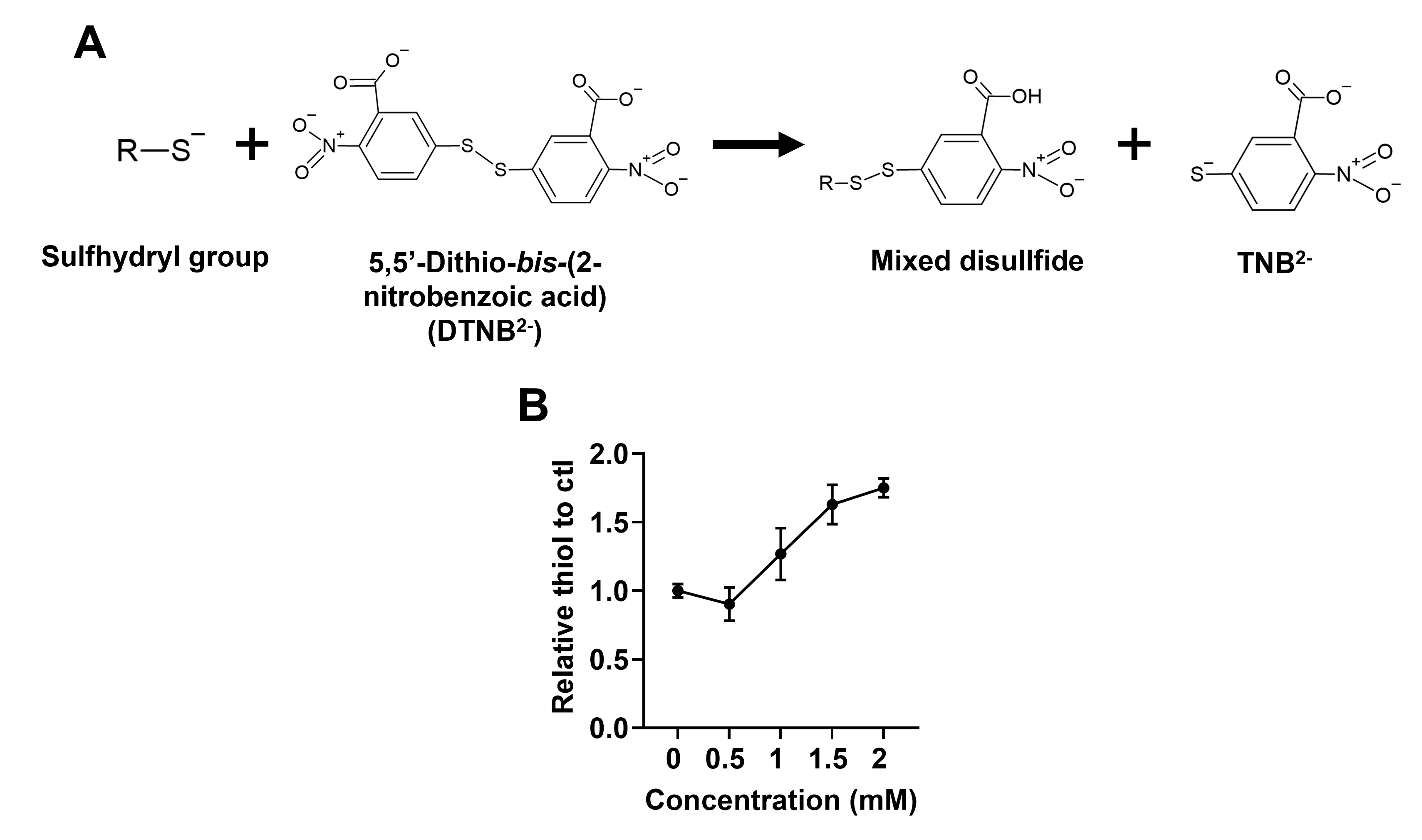

Supplement: Supplementary 1 — Figs. S1 to S6 Table S1 [file bmr.0004.f1.zip › Supplementary Figure S2.png]

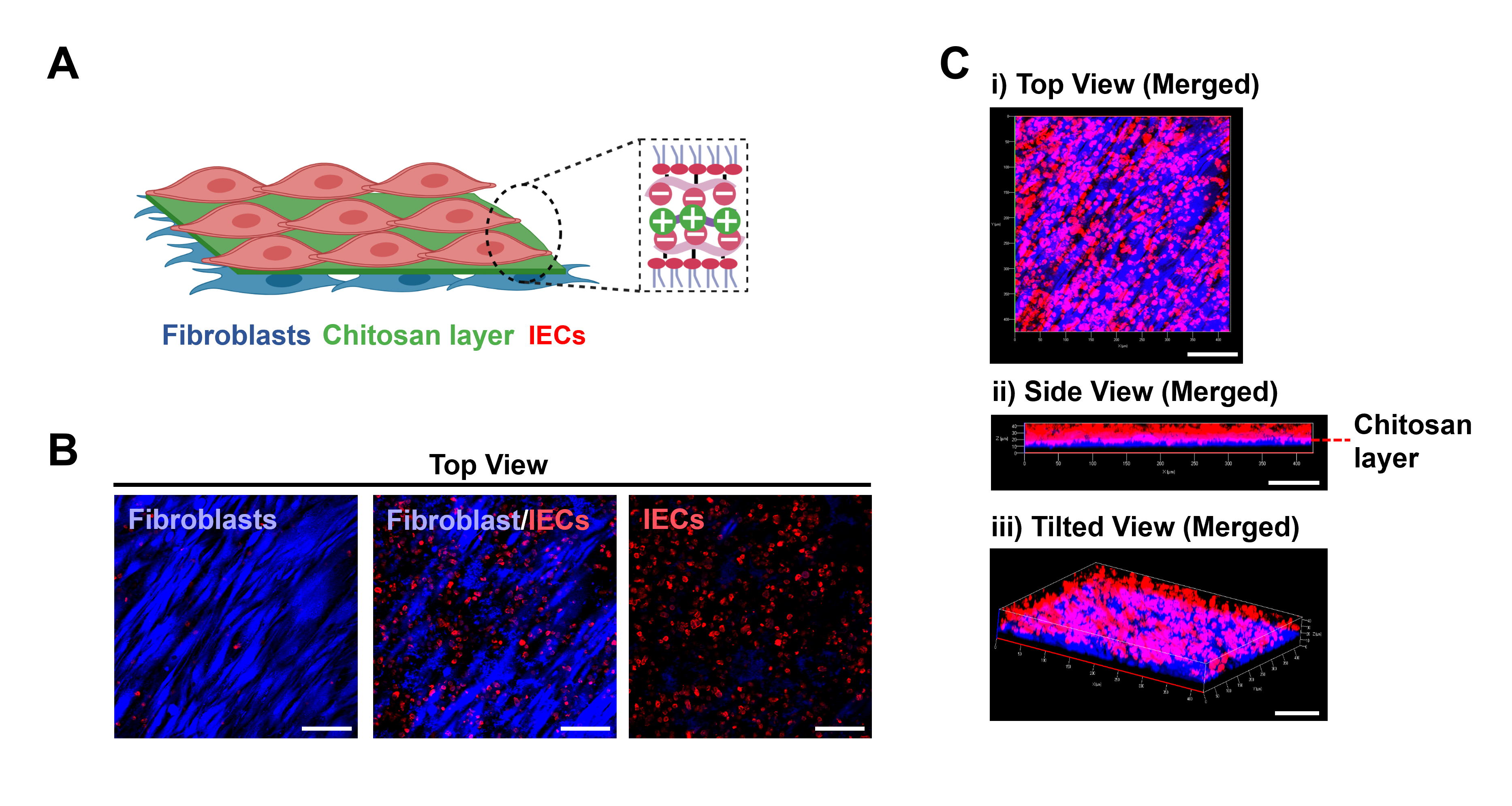

Supplement: Supplementary 1 — Figs. S1 to S6 Table S1 [file bmr.0004.f1.zip › Supplementary Figure S3.png]

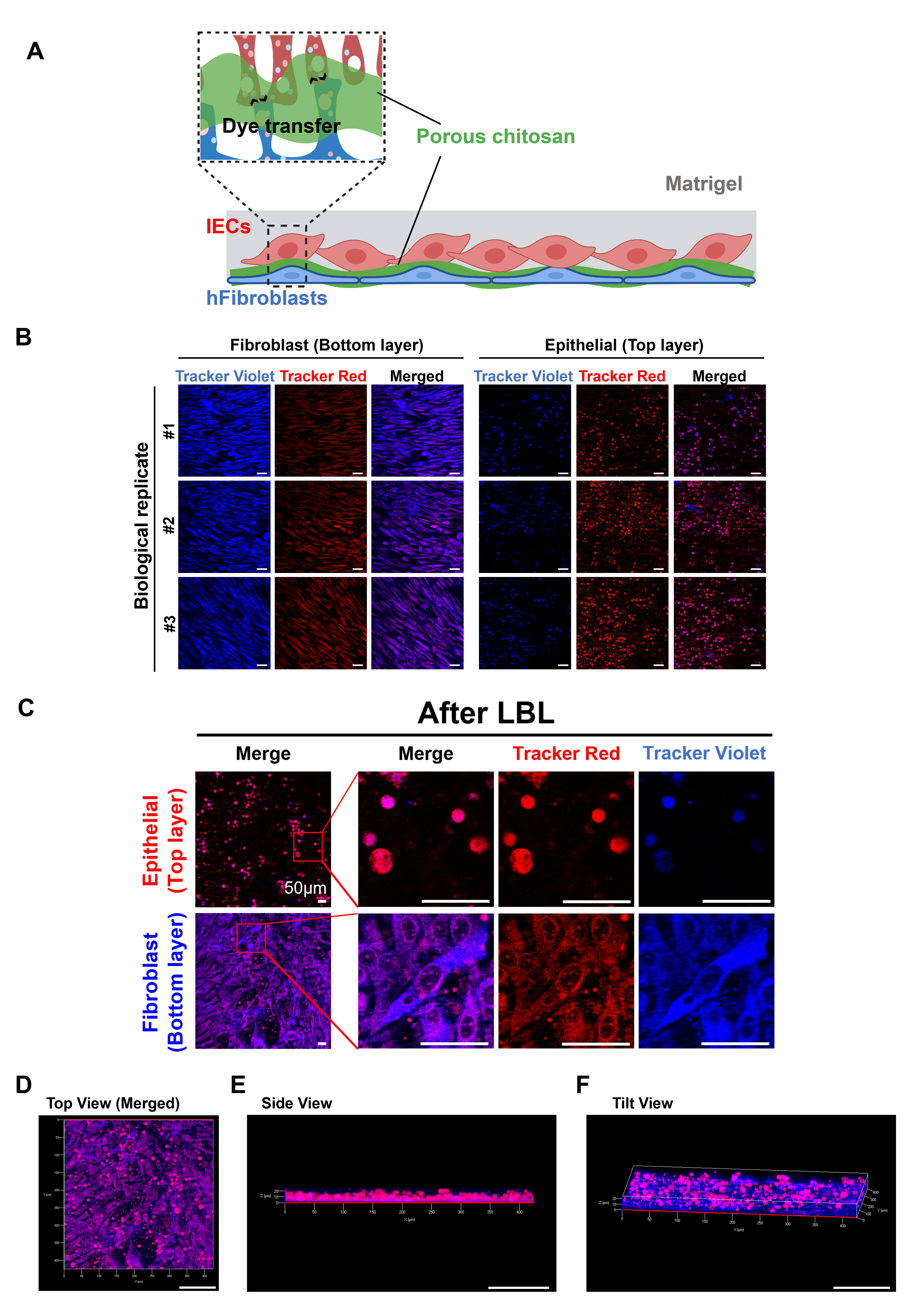

Supplement: Supplementary 1 — Figs. S1 to S6 Table S1 [file bmr.0004.f1.zip › Supplementary Figure S4.tif]

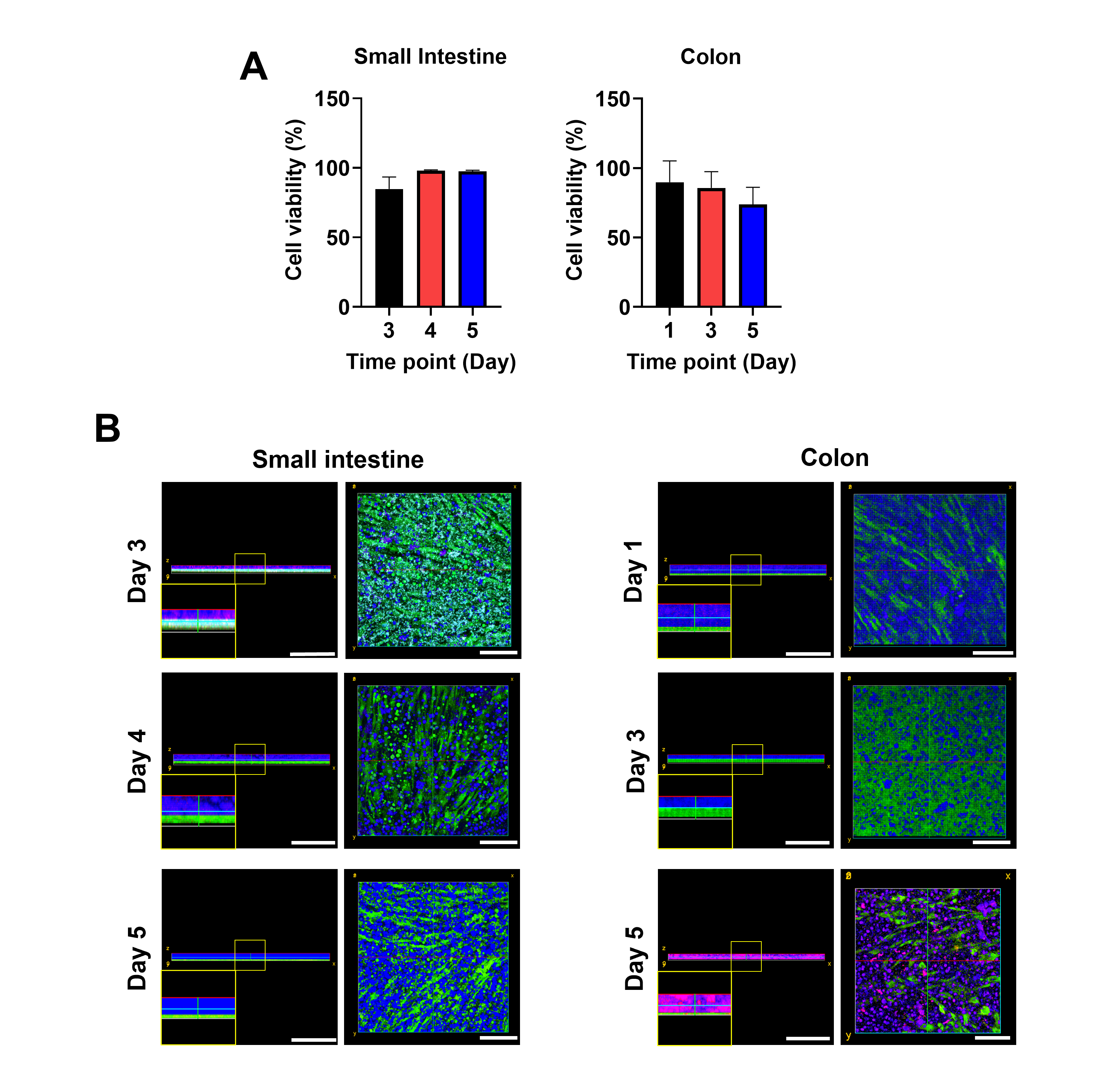

Supplement: Supplementary 1 — Figs. S1 to S6 Table S1 [file bmr.0004.f1.zip › Supplementary Figure S5.png]

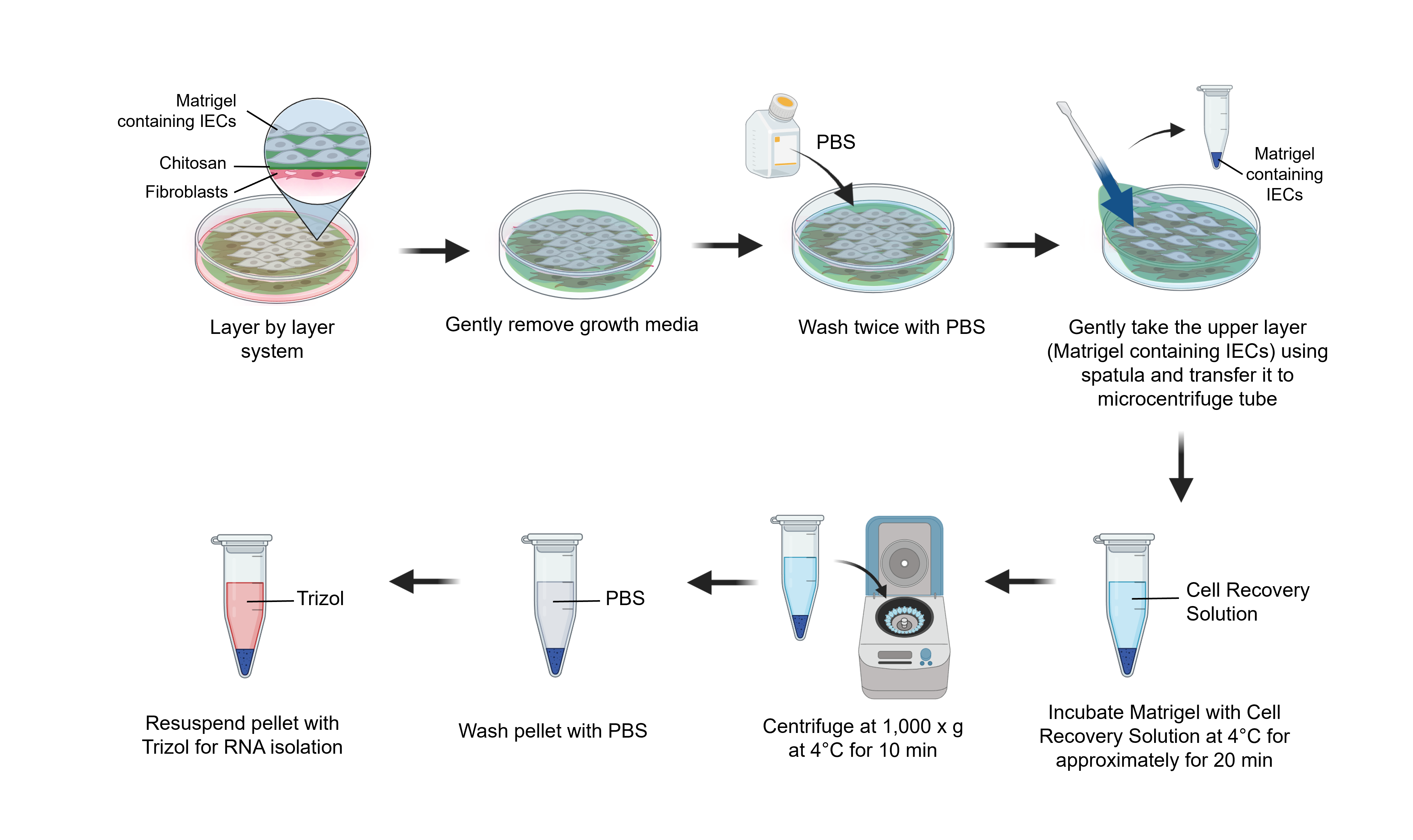

Supplement: Supplementary 1 — Figs. S1 to S6 Table S1 [file bmr.0004.f1.zip › Supplementary Figure S6.tif]

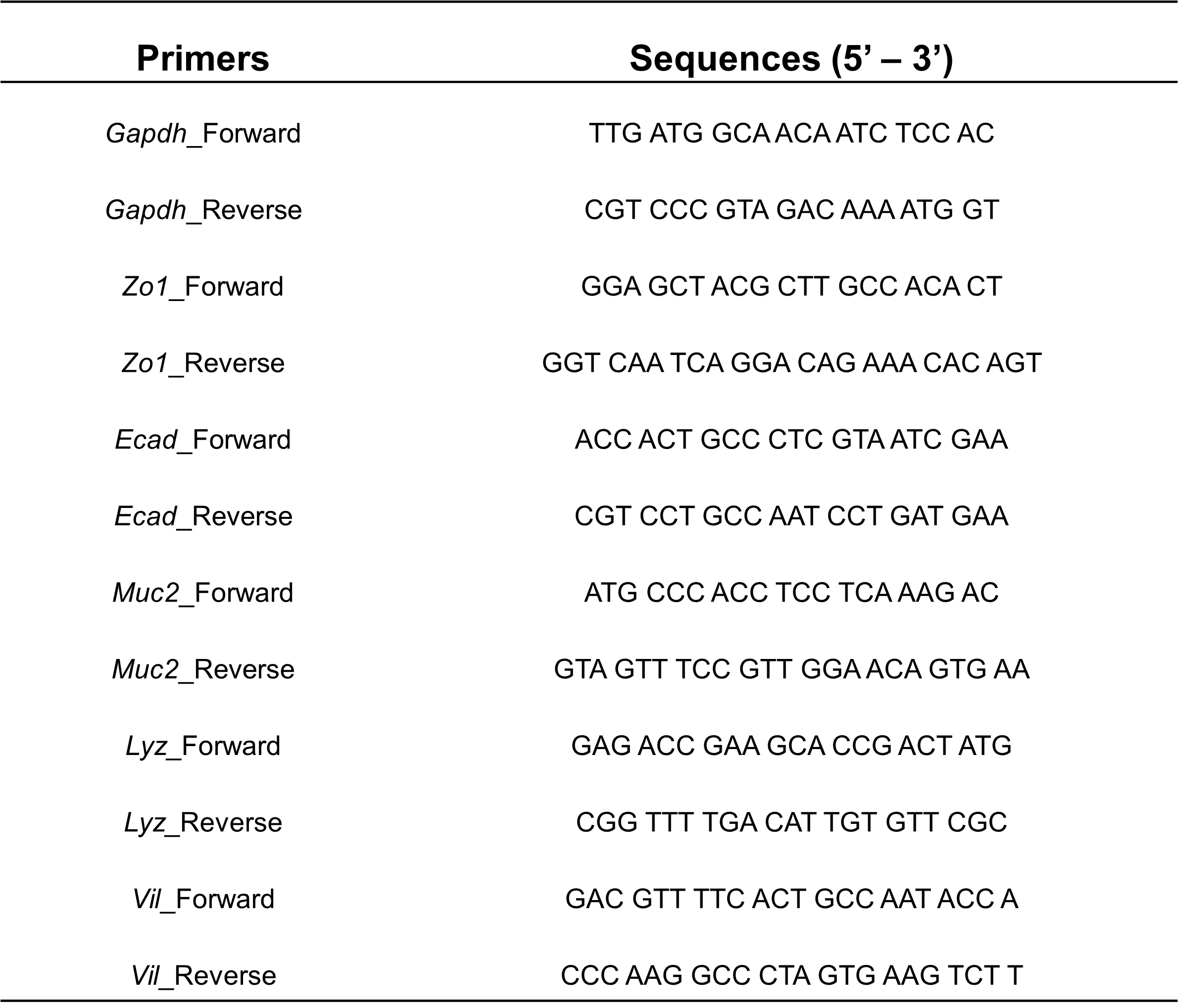

Supplement: Supplementary 1 — Figs. S1 to S6 Table S1 [file bmr.0004.f1.zip › Supplementary Table S1.tif]
